# Meaning-making following sexual abuse: a scoping review and meta-synthesis

**DOI:** 10.3389/fpsyg.2026.1798844

**Published:** 2026-03-24

**Authors:** Geert E. Smid, Jonna Lind, Renske Kruizinga

**Affiliations:** 1 ARQ National Psychotrauma Centre, Diemen, Netherlands; 2 Department of Meaningful Living and Chaplaincy Studies, University of Humanistic Studies, Utrecht, Netherlands; 3 Department of Spiritual Care & Department of Anesthesiology, Pain and Palliative Medicine, Radboud University Medical Centre, Nijmegen, Netherlands

**Keywords:** child sexual abuse, meaning-in-life, mental, posttraumatic growth, rape, recovery, spiritual, trauma

## Abstract

**Background:**

Exposure to adult sexual assault and child sexual abuse leaves survivors with questions about meaning and the process of meaning-making following sexual abuse may influence survivors’ recovery. Insight in meaning-making following sexual abuse and survivors’ perceptions of the role of meaning-making in recovery informs professionals involved in their care.

**Objective:**

This scoping review and meta-synthesis of qualitative, mixed methods, and review studies aims to explore survivors’ experiences of meaning-making following sexual abuse.

**Method:**

We searched six databases: PsycInfo, Ovid Medline ALL, Ovid Evidence-Based Medicine Reviews, PTSDpubs, and Web of Science, and screened 2,158 deduplicated references for inclusion. We followed PRISMA 2020 guidelines for reporting of the systematic search. We integrated findings of the included studies using reflexive thematic analysis.

**Results:**

Seventy-four studies were included in the review. Methodological approaches included selecting participants based on experiencing resilience and recovery following sexual abuse or participants receiving professional support, and focusing on the role of time, specific survivor actions, specific abuse or post-abuse contexts or cultural contexts. Twelve themes were generated, reflecting experiences of meaning-making following sexual abuse: reframing the abuse, moving from shame towards self-acceptance, understanding one’s own beliefs, reorienting towards religion and spirituality, helping others, connecting with a peer group, building supportive relationships, a secret (not) to be disclosed, recognizing positive change, engaging in activism, honoring strengths and hope, and living with triggers. We grouped the themes into three overarching themes: a changed self, reshaping relationships with others, and mapping out a future self and world.

**Discussion:**

Experiences of meaning-making involve turning points and transitions, e.g., from receiving to providing support, from retelling to reframing, and deliberate acts of self-care. Our findings suggest that the process of meaning-making following sexual abuse operates at existential, cognitive, emotional, motivational, social, and bodily levels. Attending to these multilayered meanings can facilitate interdisciplinary collaboration and expand professionals’ insight into their roles and responsibilities in trauma-informed care. Efforts to prevent sexual abuse and mitigate its long-term effects should include strengthening peer and community support, fostering cultural connectedness, and promoting social change.

## Introduction

The WHO defines sexual violence as “any sexual act, attempt to obtain a sexual act, unwanted sexual comments or advances, or acts to traffic, or otherwise directed against a person’s sexuality using coercion, by any person regardless of their relationship to the victim, in any setting including but not limited to home and work” (Krug et al., 2002, p. 149). Various terms are used to describe specific forms of sexual violence. Sexual violence towards children is often named child sexual abuse (CSA) and may be associated with physical abuse and emotional neglect (Krug et al., 2002). To indicate sexual violence towards adults, commonly used terms include sexual assault or rape. Victims-survivors of sexual abuse are mostly women (Krug et al., 2002). The term sexual abuse will be used throughout this article to indicate all forms of sexual violence.

Exposure to sexual abuse can have far-reaching negative consequences in many areas of life and poses a serious threat to mental health; mental health conditions associated with sexual abuse include post-traumatic stress disorder (PTSD), bipolar, depressive, and anxiety disorders, eating disorders, obsessive-compulsive disorder, sleep disorders, suicidality, and substance use disorders (Dworkin et al., 2017; Hailes et al., 2019). Compared to other potentially traumatic events capable of causing symptoms of PTSD, the risk of developing PTSD following sexual abuse is high (Liu et al., 2017). In addition, sexual abuse can have a profound impact on survivors’ beliefs and views of the world or themselves (Herman, 1992), through the meaning survivors attribute to their experiences.

Attribution of meaning following potentially traumatic events can be conceptualized as a multifaceted process, encompassing sense-making, evaluating the broader implications of the events, and reappraisal or reframing through both intra-individual and interpersonal negotiation (Bonanno, 2013; Park et al., 2012; Park and Ai, 2006). If unexpected experiences occurred under violent or accidental, “meaningless” circumstances, questions of meaning often present themselves in complex, urgent and distressing ways.

Different operational definitions of meaning-making have been used in research (Brandstätter et al., 2012; Martela and Steger, 2016). Park (2010) distinguishes between global and situational meaning. Global meaning is rooted in people’s experiences and fundamental beliefs concerning themselves, the world, their place in the world and their sense of purpose, goals and values and involves subjective feelings about the meaning of life which arises from one’s actions and is directed towards future goals. Situational meaning refers to the way people understand, construct, or attribute meaning to a specific event. Situational meaning is related to global meaning, in the context of a particular situation, and influences one’s interpretation and subsequent reactions to that situation. Situational meaning encompasses the meanings attributed to these experiences.

The process of meaning-making following exposure to sexual abuse can contribute to recovery (Park and Ai, 2006), resilience, i.e., the capacity to bounce back or to continue living a purposeful life after experiencing hardship and adversity (Bonanno, 2004), and posttraumatic growth, i.e., the experience of positive change after the occurrence of a highly stressful or challenging life crisis (Tedeschi and Calhoun, 2004). Understanding how individuals make meaning from sexual abuse can inform interventions and guide the development of programs aimed at enhancing resilience in survivors. Therefore, with this meta-synthesis, our aim is to integrate findings from empirical, qualitative studies exploring meaning-making following sexual abuse as it is experienced by survivors. The central question addressed here is: what meanings do survivors of sexual abuse attribute to their experiences of sexual abuse and how do they perceive the influence of meaning-making on their recovery?

## Methods

### Search strategy

Aiming to integrate qualitative evidence, we chose to perform a scoping review and meta-synthesis. We searched for publications exploring the meaning-making process following sexual abuse and how survivors of sexual abuse experience the role of meaning-making in their recovery. The PRISMA scoping reviews checklist (Tricco et al., 2018) was used to ensure correct reporting in line with the PRISMA 2020 framework (Page et al., 2021). This scoping review updates and extends a previously published master’s thesis that was supervised by the authors (van der Werf, 2022; see Acknowledgement) from which we adopted the systematic search strategy. We did not register the review protocol.

On April 6, 2022, we searched for existing reviews and protocols on the topic in PROSPERO, the Campbell Collaboration online library, Open Science Framework (OSF), JBI Evidence Synthesis, EBM Reviews – Cochrane Database of Systematic Reviews (Ovid), and Google Scholar and found none. On April 20–22, 2022; August 2, 2023; February 13, 2024; and July 1–2, 2025, we searched the following six databases: PsycINFO, Ovid Medline ALL, Embase, Ovid Evidence-Based Medicine Reviews (Cochrane DSR, ACP Journal Club, DARE, CCA, CCTR, CMR, HTA, and NHSEED), PTSDpubs and Web of Science. Search terms were grouped into clusters: *Population* – Women (set 1) who have experienced sexual abuse (set 2); *Outcome* – Meaning-making: meaning-making (set 3), goals and personal values (set 4), world view, ideology, religion (set 5), resilience (set 6), coping (set 7), and recovery (set 8); *Study Design*: Systematic and other reviews, meta-analyses, guidelines (set 9), RCTs (set 10), trials (set 11), case studies (set 12), qualitative studies (set 13), and other empirical studies (set 14). These clusters were combined using Boolean operators (set 15), without restrictions on publication year, language and/or similar parameters. Results were imported into Endnote and deduplicated (Bramer et al., 2016; Bramer and Bain, 2017).

Supplementary Table 1 provides full details of the search strategies. Supplementary Table 2 shows the number of references retrieved through each search system and the number of duplicates. Supplementary Table 3 shows the Preferred Reporting Items for Systematic reviews and Meta-Analyses extension for Scoping Reviews (PRISMA-ScR) checklist.

### Study selection

To screen and select the 2,158 retrieved and deduplicated references, we used Rayyan, an online program for screening, collecting, and theming references (Ouzzani et al., 2016). Search results were screened by two researchers and disagreements were solved by consensus. After screening, 74 reports met criteria and were included. We additionally searched included review studies in December 2025 for larger qualitative studies (*N* > 20) that fit our inclusion criteria – this yielded an additional 2 included studies.

**Figure 1:**
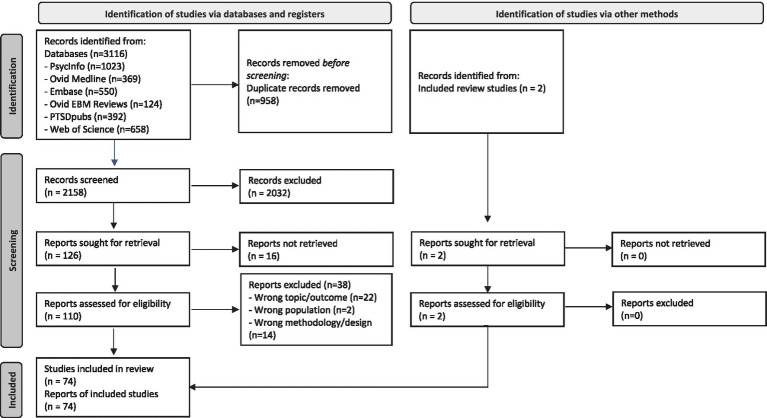
PRISMA flow chart of literature search and selection.

We selected eligible studies based on the study population, outcomes, and study design to standardize the appraisal of literature, increase transparency and reduce potential bias. Because we focused on meaning-making as a process rather than an intervention, we did not use the intervention/ comparison elements of the PICOS framework (Methley et al., 2014). Thus, records were eligible if they met the following inclusion criteria: the *population* consisted of adults (primarily women) who have experienced sexual abuse; the *outcome* included meaning-making following sexual abuse; the *study design* was empirical, including case studies, qualitative or mixed methods studies (specifically studies integrating quantitative and qualitative research questions, methods, and findings; Matović and Ovesni, 2023), or systematic, integrative, and scoping reviews and meta-analyses. Exclusion criteria were as follows: the study population consisted of only respondents younger than 18 years; the outcome was not about meaning-making following sexual abuse; the study design was not empirical or not directly from survivors of sexual abuse or only based on quantitative data. We limited selected publication types to journal articles, journal chapters and dissertations and excluded research protocols, books, and book reviews. Figure 1 shows a PRISMA diagram for an overview of the process.

### Characteristics of included studies

The selected articles were divided into primary studies and review studies. From primary studies, we abstracted the following data: study name (author/ publication year), country where the study was performed, sample size (N), participant gender (female, male, nonbinary or other), participant age range and mean age, type of data collected, type of qualitative analysis, type of sexual abuse, and additional participant selection criteria/ research focus. From review studies, we abstracted the study name (author/ publication year), country where the study was performed, number of included studies (k), type of review, inclusion criteria studies, and specific focus of the review.

### Synthesis of qualitative findings

After the selection of the final articles, we conducted reflexive thematic analysis (Braun and Clarke, 2006, 2021, 2024) to generate common and overarching themes, ideas, and patterns. As researchers exploring meaning-making among survivors of sexual abuse, we acknowledge that our own identities, perspectives, and positionalities inevitably influence the research process. Our perspectives comprise various academic and professional backgrounds (psychology, psychiatry, and spiritual care) as well as involvement in research with and/ or providing care for survivors of domestic violence and sexual abuse. A multidisciplinary, biopsychosocial-spiritual framework (e.g., Carey and Hodgson, 2018) informed our analysis. We aimed to include studies from different research disciplines with different epistemological foundations to enhance the transferability of the findings. To enable in-depth understanding of meaning-making and *crystallization*, we gathered multiple types of data that employed various methods, involved multiple researchers and numerous theoretical frameworks (Tracy, 2010).

We appraised the quality of the included primary studies by reviewing the reporting of elements of research procedures and abstracting data related to study methodology (cf. Carroll and Booth, 2015), informed by the values of reflexive thematic analysis (Braun and Clarke, 2024). To accommodate the diversity of theoretical assumptions and procedural practices within qualitative analysis, we did not apply a standardized quality assessment, in line with recommendations for qualitative meta-synthesis and reflexive thematic analysis (Braun and Clarke, 2024; Carroll and Booth, 2015).

The analysis involved immersion in and familiarization with the included studies and creating an overview table detailing research design, methodology, demographic data, types of sexual abuse, and main study findings. We generated succinct codes based on reading and re-reading the studies that captured features of the data that were relevant to addressing the research question. Patterns of meaning and recurring concepts across studies were evoked in initial themes directed by the content of the data. We collated data relevant to each candidate theme to review the viability of each candidate theme. Subsequently, we developed themes further by splitting two initial themes and adding another theme. We repeatedly refined, reviewed and revised the themes. Collaborative discussions ensured rigor, reflexivity, and multiple perspectives in the interpretation of findings, with researchers acting as *critical friends* who listen and offer critical feedback (Smith and McGannon, 2018). The final stages of the analysis consisted of naming of the themes and writing down an integrated narrative, describing the study findings.

## Results

### Characteristics of included studies

We included 74 studies that provided insight into the process of meaning-making following sexual abuse. Of the 74 studies, 69 were primary studies and 5 were literature reviews. Figure 1 shows the PRISMA flow chart summarizing the search. Table 1 shows an overview of the included primary studies. Primary studies reported about a total of 1,395 participants (1,034 women), ages when participating ranging from 13 to 81, with time since the abuse ranging from weeks to decades. Studies were from 12 countries, the large majority from the US, and covered a wide range of cultural identities, including ethnic (e.g., indigenous Māori (Harrison and Le Grice, 2023), Murega women from eastern DRC (Maisha et al., 2024)) and gender identities, and were published between 1992 and 2025.

**Table 1:** Primary studies: methodological aspects.

Study name	Country	N	Gender (n/%)	Age	Data collected	Analysis
Anderson and Hiersteiner (2008)	US	27	25F, 5M	25–68 (43.6)	Semi-structured interviews	Grounded theory
Arias and Johnson (2013)	US	10	10F	44–56 (49)	Semi-structured interviews	Grounded theory
Attrash-Najjar and Katz (2025)	Israel	14	10F, 4 M	29–65	Semi-structured in-depth interviews	Thematic
Bainbridge (2022)	UK	1	1F	n.r.	Auto-ethnography: journalling	Thematic, narrative
Barker et al. (2023)	UK	66 [7]	53 [5]F, 11[2] M, 1 nonbinary	25–65+	Survey [and follow-up interview]	Thematic
Bennett (2024)	US	9	9F	31–67	Semi-structured interviews	Grounded theory
Beveridge and Cheung (2004)	US	1	1F	29	Case description	Phased therapy framework
Bogar and Hulse-Killacky (2006)	US	10	10F	30+	Semi-structured interviews	Phenomenological approach
Brown et al. (2024)	US	91	6F, 85 unknown	35–67 (61.4)	De-identified correspondence	Thematic
Chouliara et al. (2014)	UK	22	n.r.	18+	Semi-structured interviews	Interpretative phenomenological
Dean (2022)	US	6	6F	22–48	Semi-structured interviews	Interpretative phenomenological
Dodgson (1996)	US	8	8F	27–46	Semi-structured interviews	Thematic
Draucker et al. (2011)	US	95	48F, 47 M	18–62	Semi-structured interviews	Grounded theory
Draucker (1992)	US	11	11F	20–64	Unstructured interview	Interpretative phenomenological
Driessen et al. (2024)	US	17	14F, 1M, 1 nonbinary, 1 two-spirit	20–50	Semi-structured in-depth interviews	Thematic
Duma et al. (2007a)	South Africa	10	10F	18+	In-depth interviews at 4 times	Grounded theory
Duma et al. (2007b)	South Africa	10	10F	18+	In-depth interviews at 4 times	Grounded theory
Dussault et al. (2024)	Canada	21	45.5%F, 54.5%M	25–66 (45.23)	Semi-structured interviews	Content
Ellis and Wieling (2024)	US	14	7F, 1 questioning, 3 genderqueer, 2 nonbinary, 1 demigirl	21–55	Semi-structured interviews (life review)	Thematic
Fawcett and Shrestha (2016)	UK	5	4F, 1M	n.r.	Blogs written by survivors of sexual violence	Thematic
Fayaz et al. (2025)	India	7	7F	18–26	Semi-structured interviews	Thematic
Gomez and Gobin (2024)	US	37	37F	18–30	Open-ended text responses to Cultural Betrayal Trauma Theory prompt	Thematic
Gueta et al. (2020)	Israel	14	14F	23–63 (34)	Semi-structured interviews	Interpretative phenomenological
Guzmán-Díaz and Trujano Ruiz (2023)	Mexico	4	4F	37–47	Semi-structured interviews	Dialogical narrative
Hall (2000)	US	3	3F	22–47	Open-ended interviews	Narrative, thematic
Hall (2003)	US	55	55F	22 + (35)	Open-ended interviews	Narrative, thematic
Harrison and Le Grice (2023)	New-Zealand	17	14F, 2M, 1 two-spirit	36–68	Face-to-face interviews	Narrative, bricolage approach
Harvey et al. (2000)	US	3	3F	42, 36, 28	Open-ended interviews	Narrative
Healicon (2012)	UK	2	2F	n.r.	In-depth interview	Narrative
Hegarty et al. (2022)	UK	10	10f	26–60 (43.9)	Transcripts of 21 group sessions	Thematic
Houg (2008)	US	14	14F	25–59 (37)	Open-ended interviews	Grounded theory
Houseknecht (2023)	US	2	2F	51, 55	Semi-structured interviews	Thematic
Knapik et al. (2008)	US	50	27F, 23M	18–62	Open-ended interviews	Grounded theory
Krayer et al. (2015)	UK	30	n.r.	18+	Biographical narrative interview	Thematic
Leahy et al. (2003)	Australia	10	5F, 5M	21–47 (33.1)	Semi-structured interviews	Thematic
Lebowitz and Roth (1994)	US	15	15F	19–52 (28.6)	Unstructured interview	Thematic
Maisha et al. (2024)	Democratic Republic of Congo	10	10F	18+	Semi-structured interviews	Thematic
Maxwell et al. (2024)	US	10	10F	21–38 (34.5)	In-depth interview	Theory-testing deductive
Millar and Stermac (2000)	Canada	6	6F	(39.7)	Semi-structured interviews	Grounded theory
Mountz et al. (2024)	US	13	6F, 5 nonbinary, 1 nonbinary/ female, 1 trans man	20–25	Semi-structured interviews	Critical thematic
Mughal et al. (2024)	US	17	17F	18–26	Photo-experiencing and Reflective Listening interviews	Thematic
Murphy et al. (2009)	US	12	12F	19–49	Semi-structured interviews	Heideggerian phenomenological
Olson (2015)	US	12	12F	19–61	Semi-structured interviews	Grounded theory
Paige and Thornton (2015)	Australia, New Zealand	35	31F, 4M	19–56+	Semi-structured interviews	Interpretative phenomenological
Panepinto (2004)	US	3	3F	18–45 (28.9)	Multidimensional Recovery and Resiliency Interview with added open-ended prompts	Grounded theory, process coding
Parrish-Martin (2023)	US	9	9F	23–53 (34.4)	Semi-structured interviews	Interpretative phenomenological
Phanichrat and Townshend (2010)	UK	7	4F, 3M	23–57 (45.14)	Semi-structured interviews	Interpretative phenomenological
Pooler and Barros-Lane (2022)	US	159	159F	(47.0)	Internet-based survey	Thematic framework
Preston et al. (2023)	US	6	6F	38–63	Semi-structured interviews	Thematic
Ramakrishnan (2024)	US	12	12F	23–31 (26.6)	Semi-structured interviews	Thematic
Saxena and Oosterhoff (2023)	India	5	5F	23–35	Semi-structured interviews	Thematic, autoethnographic
Sicilia et al. (2024)	Spain	7	3F, 4 M	38–64 (49.3)	Semi-structured in-depth interviews	Thematic
Simon et al. (2010)	US	108	84F, 24 M	13–23	Semi-structured interviews	Structural coding
Singh et al. (2013)	US	10	10F	18+	Semi-structured interviews	Thematic
Sinko (2019)	US	24 [17]	24 [17]F	18–26 (22.06)	Semi-structured interviews [and photo-voice]	Grounded theory
Smith and Kelly (2001)	US	7	7F	22–60	Open-ended interviews	Thematic
Stidham et al. (2012)	US	121	64F, 57 M	18–62	Semi-structured interviews	Grounded theory
Stoner-Harris (2024)	US	1	1F	18+	Personal narrative	Auto-ethnography
Strauss Swanson and Szymanski (2020)	US	16	13F, 2M, 1 genderqueer	20–73 (39.44)	Semi-structured interviews	Thematic
Thomas and Hall (2008)	US	27	27F	29–79 (48.9)	Open-ended interviews	Interpretative narrative
Tummala-Narra et al. (2023)	US	16	16F	20–38 (27.13)	Semi-structured interviews	Content, integrative contextual framework
van Wormer and Berns (2004)	US	9	9F	33–79	In-depth interviews	Narrative
Vazquez (2019)	US	6	6F	32–62 (44.5)	Semi-structured interviews	Grounded theory
Vilenica et al. (2013)	Australia	2	2F	44 and 52	Semi-structured interviews	Interpretative phenomenological
Walker-Williams and Fouché (2018)	South Africa	8	8F	18–36 (25)	Transcripts of 10 group sessions	Thematic
Wilson (2020)	US	7	7F	39–64	Focus group	Theological underpinnings
Winterstein et al. (2023)	Israel	11	11F	60–81 (68)	Biographical narrative interviews	Thematic
Wright et al. (2007)	US	60	60F	(38.8)	Text responses to open-ended questions	Thematic
Yih (2024)	Hong Kong	8	8F	18+	Semi-structured interviews	Interpretative phenomenological

F: female; M: male.

To obtain an overview of methodological approaches across the included primary studies, we categorized the research focus and/ or participant selection criteria that were applied within the studies in addition to having experienced sexual abuse. We found the following categories: studies selecting participants based on experiencing resilience and recovery following sexual abuse; studies that selected participants receiving professional support; studies focusing on the role of time; studies of specific survivor actions, e.g., disclosure of the sexual abuse; studies of specific abuse contexts, e.g., abuse by clergy; studies of specific post-abuse contexts, e.g., substance abuse; studies focusing on cultural contexts of the sexual abuse, e.g., cultural or gender identity. Table 2 shows the primary studies within each of these categories. Table 3 summarizes the 5 included review studies.

**Table 2:** Primary studies grouped by participant selection criteria/ research focus.

Study name	Type of abuse	Additional participant selection criteria/ research focus
Resilience and recovery		
Arias and Johnson (2013)	CSA	Perceiving themselves as doing relatively well in most spheres of life
Bainbridge (2022)	Internet teenage sexual abuse	Being a survivor “wounded” researcher and therapist
Bennett (2024)	CSA	Being presently involved in committed relationships
Bogar and Hulse-Killacky (2006)	CSA	Endorsing a satisfactory current life situation and believing that their lives had meaning
Chouliara et al. (2014)	CSA	Experiencing a recovery journey
Draucker (1992)	Incest	Experiencing some healing or sharing their own healing experiences in some public context
Draucker et al. (2011)	CSA	Experiencing some healing
Harvey et al. (2000)	CSA, other sexual violence	Taking part in study of recovery and resiliency in trauma survivors
Houg (2008)	CSA	Willing to be interviewed about the role of spirituality in the ongoing recovery process
Knapik et al. (2008)	Sexual violence	Using spirituality in response to sexual violence
Leahy et al. (2003)	CSA	Active competitors in the organized competitive sports system
Olson (2015)	Sexual assault at age 14+	Not currently involved in legal proceedings related to the assault
Panepinto (2004)	Sexual assault at age 16+	Feeling that they had recovered
Phanichrat and Townshend (2010)	CSA	Considering themselves recovered or moved on
Smith and Kelly (2001)	Sexual assault	Recovering or having recovered from the experience of rape
Thomas and Hall (2008)	CSA	Thriving, community dwelling CSA survivors
Vilenica et al. (2013)	Sexual assault	Consider themselves to be healed or well into their healing journey
Receiving professional support		
Anderson and Hiersteiner (2008)	CSA	Attending adult survivor support groups
Beveridge and Cheung (2004)	Sexual molestation	Taking part in therapy at a specialized family-based treatment facility
Hegarty et al. (2022)	CSA	Attending a specialist out-patient psychological trauma service
Houseknecht (2023)	CSA	Utilizing individual counseling on at least a bi-weekly basis and have addressed CSA
Parrish-Martin (2023)	Sexual assault	Completing 7 sessions of Creative Arts Personal Growth Group with Centering Prayer
Walker-Williams and Fouché (2018)	CSA	Participation in a collaborative strengths-based group intervention program
Role of time		
Duma et al. (2007a)	Sexual assault	Recent (<1 week) assault
Duma et al. (2007b)	Sexual assault	Recent (<1 week) assault
Krayer et al. (2015)	CSA	focus on narratives in and through time
Murphy et al. (2009)	Sexual assault	Within 5 years before the data collection
Simon et al. (2010)	CSA	Participation in longitudinal study of the consequences of CSA
Winterstein et al. (2023)	Intrafamilial CSA	Older adults (60+), CSA between ages 4–10
Wright et al. (2007)	CSA	Adult mothers
Survivor actions		
Attrash-Najjar and Katz (2025)	CSA	Engaging in anti-sexual assault activism
Barker et al. (2023)	CSA	Sharing impacts of abuse and recommendations for change
Brown et al. (2024)	Sexual violence	Responding to public testimony to a high-profile case with unsolicited personal correspondence
Dodgson (1996)	CSA	Having disclosed about being survivors of CSA since the age of 18 to at least one person
Driessen et al. (2024)	Sexual victimization at age 14+	Disclosing online about their sexual victimization
Fawcett and Shrestha (2016)	Sexual violence	Writing a blog on the experience of victimization and recovery
Gueta et al. (2020)	Sexual assault	Publicly disclosing their sexual assault online
Healicon (2012)	Sexual violence	Confident to talk about their experiences
Maxwell et al. (2024)	Sexual trauma	Having at least one tattoo
Paige and Thornton (2015)	Intrafamilial CSA	Contacting the parent who had abused them
Strauss Swanson and Szymanski (2020)	Adult sexual assault	Involvement in anti–sexual assault activism
Specific abuse contexts		
Dussault et al. (2024)	CSA	Experiencing CSA and psychological maltreatment
Maisha et al. (2024)	Sexual violence	War-related rape survivors, Murega members of the Warega tribe in Mwenga, eastern DRC
Mughal et al. (2024)	Nonconsensual sexual encounters	While enrolled as an undergraduate student
Pooler and Barros-Lane (2022)	Sexual abuse at age 16+	By clergy
Preston et al. (2023)	Sexual trauma	In military context
Ramakrishnan (2024)	Sexual assault	Reporting experiencing unwanted arousal during rape
Sicilia et al. (2024)	CSA	By clergy
Sinko (2019)	Unwanted sexual experience	While enrolled as an undergraduate student
van Wormer and Berns (2004)	Sexual abuse (child or adult)	By clergy
Specific post-abuse contexts		
Dean (2022)	CSA	Experiencing commercial sexual exploitation
Hall (2000)	CSA	In recovery from substance misuse
Hall (2003)	CSA	Having a history of substance misuse
Millar and Stermac (2000)	CSA	In recovery from alcohol and/or drug addiction
Cultural contexts		
Ellis and Wieling (2024)	Sexual assault	Belonging to a sexual minority
Fayaz et al. (2025)	CSA, other sexual violence	Living in a patriarchal society
Gomez and Gobin (2024)	Sexual violence	Self-identified Black women
Guzmán-Díaz and Trujano Ruiz (2023)	CSA	Engaging in therapy
Harrison and Le Grice (2023)	Intrafamilial CSA	Indigenous Māori
Lebowitz and Roth (1994)	Adult or adolescent sexual assault	Survivors of completed rape
Mountz et al. (2024)	CSA, sexual assault	Black LGBTQ + girls, femmes, transgender and nonbinary young people
Saxena and Oosterhoff (2023)	Intimate partner violence	Living in a patriarchal society
Singh et al. (2013)	CSA before age 12	African American woman
Stidham et al. (2012)	CSA	Experiencing some healing
Stoner-Harris (2024)	CSA	Black female survivor of CSA
Tummala-Narra et al. (2023)	CSA	Having one or more parents born in Mexico, living in the US from age 0–12
Vazquez (2019)	CSA	Self-identifying as Latino/ Hispanic, using creative arts, religiosity/ spirituality to cope
Wilson (2020)	CSA	Relationship with a Christian faith community or church
Yih (2024)	Sexual harassment experiences	Sought support from their faith communities

**Table 3:** Review studies.

Study name	Country	*k*	Type of review	Inclusion criteria studies	Specific focus
Bryant (2024)	US	n.r.*	Narrative	Not reported	Views on trauma and recovery from decolonial and liberation psychologies
Bryant-Davis and Wong (2013)	US	56	Narrative	Not reported	Spiritual and religious coping among survivors of child abuse, sexual violence, and war
Draucker et al. (2009)	US	51	Qualitative metasynthesis	Adults’ responses to sexual violence	Responses to sexual violence of adults living in the US or Canada
van der Westhuizen et al. (2023)	South Africa	57	Scoping	Meaning making in the context of CSA	Experiences of female survivors of (child) sexual abuse
Wright and Gabriel (2018)	UK	15	Critical	Adult survivors of CSA	Adult survivors of CSA referred to individual talking therapies

k: number of included studies. *Not reported (116 references).

### Synthesis of qualitative findings

From the included studies, 12 themes were created reflecting meanings that survivors attribute to their experiences of sexual abuse: reframing the abuse, moving from shame towards self-acceptance, understanding one’s own beliefs, reorienting towards religion and spirituality, helping others, connecting with a peer group, building supportive relationships, a secret (not) to be disclosed, recognizing positive change, engaging in activism, honoring strengths and hope, and living with triggers.

### Theme 1: Reframing the abuse

By naming and reframing their experiences, survivors of sexual abuse provided meaning to their experiences. Finding the right words to describe sexual abuse enabled survivors to realize they were not complicit in the abuse (Houseknecht, 2023). Recovery from rape involved reframing the rape and redefining the self (Smith and Kelly, 2001). Cultural beliefs or constructions about women were accessed by women survivors of rape as they construe meaning to the experience of being raped. During this process, labeling the rape as rape was an important step in recovery (Lebowitz and Roth, 1994). The realization that the abuse was not the survivors’ fault led to rejecting self-blame and holding the perpetrator responsible for the rape (Panepinto, 2004). A complication in this process was unwanted arousal during sexual abuse. Female survivors of rape who reported unwanted arousal during rape experienced self-doubt and were exposed to victim-blaming by loved ones and professionals in response to the unwanted arousal (Ramakrishnan, 2024). Reframing the sexual assault narrative, e.g., as “it’s not only me, but a social phenomenon that needs to be fixed!” (Gueta et al., 2020, p. 631) was supported by public online self-disclosure of sexual assault. In women who were sexually abused by clergy as adults, mental health counseling facilitated recognizing and naming what had happened to them: “My husband believed and understood that what happened to me was abuse, NOT an affair!” (Pooler and Barros-Lane, 2022, p. 130).

Reframing the abuse involved turning points and narrative reconstruction, enabling survivors to move from powerlessness and avoidance toward agency. Stories of sexual violence shared aspects of breaking down and making meaning of the violence as a series of steps in understanding the assault (Murphy et al., 2009). *Turning points* opened possibilities to arrive at new understandings and narratives that replaced long-standing feelings of powerlessness with a new sense of agency (Harvey et al., 2000). Recovery within the first 6 months following rape consisted of stages characterized by the following themes: sexual assault trauma, awakening, pragmatic acceptance, turning point, reclaiming what was lost, defining landmarks of healing, readiness for closure, and returning to self (Duma et al., 2007a, 2007b). While low self-worth characterized CSA survivors’ earlier years, specific events or turning points led to a realization of the possibilities of change and a potential/developing self (Krayer et al., 2015). In young CSA survivors, effortful processing of abuse memories, affects, and cognitions characterized a *constructive* narrative. An engrossment in abuse related memories, affects, or cognitions in ways that impede meaning-making characterized an *absorbed* narrative. *Avoidant* narratives were characterized by a marked lack of effort to evaluate the impact of the abuse on the self or relationships (Simon et al., 2010).

### Theme 2: Moving from shame towards self-acceptance

Shame after sexual abuse fostered self-blame, guilt, secrecy, and social withdrawal, impeding recovery. Beliefs of being unworthy, guilty or hideous stemmed from deep feelings of shame and perceived guilt over the assaults, incorrectly internalizing the assaults as something they contributed to and for which they were responsible (Vilenica et al., 2013). Shame and fear of negative social reactions were reasons for nondisclosure of sexual abuse (Brown et al., 2024). Shame promoted hiding, avoiding, and withdrawing behaviors that impeded the process of rebuilding the self through accepting, connecting, reclaiming power, and creating meaning and purpose (Olson, 2015).

Attributing responsibility for the sexual abuse to perpetrators constituted an important step towards self-acceptance, which involved integration of the sexual abuse into one’s self and identity. In CSA survivors experiencing recovery, the affected self was characterized by self-blame, guilt, shame, aloneness and social stigma, while the recovering self was characterized by increasing confidence, assertiveness, ability to self-care and self-acceptance, and by embracing vulnerability (Chouliara et al., 2014). In a cognitive behavioral therapy group for adult survivors of CSA with complex PTSD symptoms (comprising re-experiencing, hypervigilance, avoidance, emotional dysregulation, negative self-concept and disturbances in relationships) shifting of self-blame catalyzed schema changes related to the self (improved self-concept), others and future (Hegarty et al., 2022). A core theme in adult survivors of incest was “constructing a personal residence,” a process that included building a new relationship with the self and attributing responsibility for the abuse to the offender, the family or society (Draucker, 1992). The realization that the abuse was not the survivors’ fault led to a rethinking of guilt, shame, and self-blame (Wright and Gabriel, 2018), fostering self-acceptance (Phanichrat and Townshend, 2010). CSA survivors who recovered from substance abuse described the development of a new self-concept, relinquishing their presentation of false fronts and connecting more fully with their authentic self (Millar and Stermac, 2000). CSA survivors who disclosed their abuse moved towards developing greater self-acceptance as they integrated their abuse survival as an important aspect of identity (Dodgson, 1996). Female undergraduate survivors of campus unwanted sexual experiences were able to pursue identified healing goals by externalizing their trauma and integrating it into their core identity (Sinko, 2019). African American women CSA survivors described integrating multiple identities as a survivor (Singh et al., 2013).

### Theme 3: Understanding one’s own beliefs

Meaning-making following sexual abuse involved examining and modifying deeply held personal and cultural beliefs. When survivors understood what beliefs they held, how these historically developed, and when they examined the validity of their beliefs, a modification of beliefs resulted: “It was just a gradual unpicking of all the parts of my life that had been built up around having been raped and looking at them one by one and seeing if I wanted to keep them” (Vilenica et al., 2013, p. 47). During therapy, unhelpful beliefs, e.g., “I must respect my elders,” could be modified through Socratic questioning, e.g., “How does this cultural belief apply to this case?” into more helpful beliefs, e.g., “It is okay to feel rage toward the perpetrator” (Beveridge and Cheung, 2004, p. 115). In a CSA survivor group therapy study, resilience and growth led to a changed life philosophy and a conscious re-evaluation of the self: “It’s a process I understand, now I know I don’t need to feel insecure any more, I don’t have to think people don’t like me anymore, but I need to evaluate myself every time” (Walker-Williams and Fouché, 2018, p. 7). Similarly, in clergy-perpetrated CSA survivors, a cognitive and ruminative introspection and therapy process led to a new comprehension of the life experience of the CSA trauma (Sicilia et al., 2024). Women recovering from adulthood substance abuse following prolonged CSA struggled to confront and relinquish their presentation of false fronts and connect more fully with their authentic self (Millar and Stermac, 2000).

Cultural beliefs were involved in attributing meaning to one’s experiences, and understanding the broader cultural context of sexual violence helped survivors reconnect with their self, others, and their community. Raped women associated steps toward recovery with recognizing and understanding that rape is a common phenomenon and becoming aware of the relationship between rape, broad cultural patterns of gendering, and one’s response to being raped (Lebowitz and Roth, 1994). In black female survivors of CSA, overcoming the attack on their personhood involved considering abuse in relation to the racism, sexism, and overarching oppression and reconnecting to culture and the ancestors (Stoner-Harris, 2024). Among adult Latina and/or Hispanic CSA survivors, the process of writing, painting, drawing, and using clay allowed them to express thoughts and feelings and experience a connection to the self, strength, and inner peace (Vazquez, 2019). Sexual minority women and nonbinary individuals’ lived experiences of growth involved new learning about the self, such as learning that, in healthy and safe relationships, one can be loved for who they are (Ellis and Wieling, 2024).

### Theme 4: Reorienting towards religion and spirituality

Spiritual and religious meaning-making may enhanced acceptance, transformation, transcendence, and growth and sometimes involved experiences of deliverance and forgiveness toward the self or the perpetrator. Religion and spirituality provided survivors with the opportunity to attribute meaning to the trauma they experienced (Bryant-Davis and Wong, 2013). Diverse culturally grounded pathways for survivors to reclaim themselves included culture as medicine (traditional cultural healers), spirituality and religiosity, and expressive arts (Bryant, 2024). Cultural and spiritual reorientation involved deepening spirituality (Walker-Williams and Fouché, 2018), disputing unhelpful religious assumptions (Beveridge and Cheung, 2004), taking part in creative arts groups (Parrish-Martin, 2023), and tattoos (Maxwell et al., 2024). Among women CSA survivors, most identified spirituality or religion as being important components of their resiliency (Bogar and Hulse-Killacky, 2006) supporting acceptance of the past and growth (Vilenica et al., 2013). Women survivors of adult sexual assault described profound changes in meaning and spirituality (Olson, 2015). In a US study among survivors who indicated the use of spirituality in response to sexual violence, *being delivered* reflected experiences of being rescued, saved, or set free from the effects of sexual violence by a spiritual being or power comprising three dimensions: spiritual connection, spiritual journey, and spiritual transformation (Knapik et al., 2008). Women CSA survivors experienced spiritual growth, transcendence, and self-acceptance caring for others (Wright et al., 2007). African American women CSA survivors transformed religious prescriptive messages about sex, sexuality, and womanhood into helpful spiritual practices, e.g., prayer (Singh et al., 2013). Women CSA survivors in recovery from substance abuse described a spiritual connection that included a positive “value” accorded to negative experiences, the church as a location for accessing God and communing with others, and a growing, mystical relationship to the natural world and the body (Hall, 2003). Spirituality helped women CSA survivors to better understand life events and allow forgiveness towards self and others (Houg, 2008). Older women survivors of intrafamilial CSA spiritually framed their abuse experiences as a platform for self-growth, and forgiveness seemed to play an important part in recovering (Winterstein et al., 2023). For religiously oriented Latinas and/or Hispanics, reflecting on their experiences of CSA facilitated awareness, empowerment, and forgiveness and was supported by religious coping, spiritual practice, and use of the expressive arts (Vazquez, 2019). Women survivors of intrafamilial CSA seemed forced to resolve the issue of forgiveness in response to imperatives to keep ties with the family and locally predominant Christian notions about the necessity to forgive in order to assure one’s own salvation, however, many women did not forgive their perpetrators (Thomas and Hall, 2008).

Spiritual struggle resulted from lack of religious support following sexual abuse or clergy involvement in the abuse. Women who were sexually abused by clergy as adults were often blamed for the abuse and ignored by people in their congregations but nevertheless reported positive beliefs regarding their ability to recover, facilitated by mental health counseling, faith in God, organizational support, and family support (Pooler and Barros-Lane, 2022). A study of posttraumatic growth in clergy-perpetrated CSA survivors showed a close relation between damage and growth; survivors generally rejected the Catholic faith and arrived at a new understanding of spirituality (Sicilia et al., 2024). Women who were seeking help years after clergy-perpetrated CSA described the loss of innocence, a period of self-blame, the loss of religious faith, immense pressure to maintain silence, recognition of the imbalance of power, and healing through outside help (van Wormer and Berns, 2004). Indian female survivors of sexual violence experienced a phase during which they felt disconnected from their religion, followed by an ability to demarcate the difference between society’s interpretation of the holy texts and their actual meaning (Fayaz et al., 2025). Women CSA survivors conveyed how their faith was lost, crushed, or redefined as they sought clarity about how the abuse they experienced impacted their faith (Wilson, 2020). In Christian women survivors of sexual violence in Hong Kong, facing a lack of pastoral or congregational support in the aftermath of their disclosure exacerbated their traumatic overwhelm and caused spiritual struggle (Yih, 2024).

### Theme 5: Helping others

Survivors of sexual abuse found meaning and purpose by engaging in benevolent acts and helping others through advocacy, prevention, mentorship, and professional roles informed by their experiences. After they experienced some healing from their experiences, many survivors of sexual abuse actively engaged in helping others (Stidham et al., 2012). Being understanding consisted of being generally kind, compassionate, understanding, or sensitive toward others (Stidham et al., 2012). Helping others included taking actions to prevent children from being abused, harmed, or treated in a unhealthy way; choosing a helping profession to help victims of abuse or to perform such roles more effectively based on their own experiences; providing guidance about how to avoid, or heal from, violence or abuse; becoming involved in advocacy organizations or groups; speaking out publicly about violence and abuse; and taking actions to stop perpetrators from abusing others by reporting the abuse or taking actions to bring them to justice (Stidham et al., 2012). By being benevolent and helping others, abuse survivors lived meaningful and contributed to collective healing (van der Westhuizen et al., 2023). CSA survivors’ sought meaning strategies through helping others: “I want to turn my negative experience into something positive. Not just for me but, perhaps, if I can help other guys to be more positive about their life” (Phanichrat and Townshend, 2010, p.72). By helping others, women survivors of adult sexual assault created meaning and purpose (Olson, 2015). Women survivors of intrafamilial CSA displayed remarkable generativity, parenting their own children well, and mentoring other young girls, especially victims of abuse (Thomas and Hall, 2008). An autoethnographic study by a survivor-therapist highlighted the value of sisterhood between female identifying survivors of sexual violence; where therapist self-disclosure was used judiciously in service of the client, the implicit “knowing” deepened the therapeutic relationship (Bainbridge, 2022).

By publicly sharing their stories, supporting others, advocating for systemic change, and engaging in relational or political processes, sexual abuse survivors fostered recovery and growth. A study of blog posts written by survivors of sexual assault found that blogging enabled them to use their negative experience to educate and support others going through a similar experience, allowing the bloggers to achieve some positive growth (Fawcett and Shrestha, 2016). In another study of public online disclosure of sexual assault, one respondent explained: “If you think about the assault story as something that you can help others with, I think it can give you strength to heal because you need something meaningful in order to live” (Gueta et al., 2020, p. 630). Survivors of military sexual trauma found ways to recover and give back to the world, using mantras such as: “Just keep going, make it a better place” (Preston et al., 2023, p. e1487). Survivors of clergy-perpetrated CSA associated their growth process with the act of taking care of others, such as acting as a protective figure for children and youth (Sicilia et al., 2024). Sexual minority women and nonbinary individuals experienced posttraumatic growth as a political, iteratively individual and relational process of queer worldmaking and a process of relating traumatic experiences to larger systems of oppression (Ellis and Wieling, 2024). CSA survivors who took part in the Truth Project about CSA in England and Wales reported several positive impacts: challenging beliefs that survivors of CSA cannot safely talk about their experiences and providing evidence of the central role survivors should have in designing services for trauma victims. The public visibility of the Truth Project enabled survivors to feel less alone in their abuse, and to develop a destigmatized way of talking about non-recent CSA (Barker et al., 2023).

### Theme 6: Connecting with a peer group

Peer support groups enabled sexual abuse survivors to share experiences, which fostered connection, meaning-making, empowerment, and posttraumatic growth. Support groups were experienced as a source of meaning and a safe place, because survivors felt supported by the experience of other people who understood them and showed empathy (van der Westhuizen et al., 2023). Adult sexual abuse survivors attending support groups indicated that developing supportive relationships was a critical element in recovery. Sharing their own story and hearing other survivors’ stories supported sensemaking, acknowledged victimization, affirmed self-worth and empowered survivors to have a voice of their own (Anderson and Hiersteiner, 2008). Women CSA survivors living in the metropolitan area of Mexico City who took part in therapy, described peers helped her to re-define her story. “I felt the others made me feel I was not the only one, nor that I was crazy, nor was weird or anything (…) not know anyone who would have been the same” (Guzmán-Díaz and Trujano Ruiz, 2023, p. 526). In a cognitive behavioral therapy group for adult survivors of childhood trauma with CPTSD symptoms, key themes centered on group solidarity, shifting self-blame, feeling safe in the present, and increased optimism for the future (Hegarty et al., 2022). In survivors of sexual assault who took part in a Creative Arts Personal Growth Group, a sense of common humanity helped to correct shaming experiences and live more genuinely with others (Parrish-Martin, 2023). In women survivors of CSA who participated in a group intervention program, posttraumatic growth included transforming wounded to healer (Walker-Williams and Fouché, 2018).

### Theme 7: Building supportive relationships

Survivors of sexual abuse who developed supportive relationships experienced safety, trust, empathy, connection, emotional well-being, and posttraumatic growth. Survivors of sexual abuse recovered through community support (Bryant, 2024), relating to important others and seeking safety (Draucker et al., 2009). Survivors of CSA found meaning in being supported by others and their environment and being able to build supportive relationships (van der Westhuizen et al., 2023). College-aged women who experienced sexual violence reported that support from formal and informal networks promoted their emotional well-being (Mughal et al., 2024). Adult CSA survivors emphasized the value of seeking support (Phanichrat and Townshend, 2010) and the significance of supportive relationships, including therapy, compassion and empathy (Arias and Johnson, 2013). While not being heard reinforced CSA survivors’ feeling cut-off from others, supportive connections with others were important drivers for the development of a more optimistic and active sense of self (Krayer et al., 2015). Adult survivors of incest engaged in a process of constructing a residence that included regulating one’s relationships with others (Draucker, 1992). In women survivors of CSA who were in committed relationships, the progression of trust-building comprised three distinct phases: awareness, attachment-repairing behaviors, and the creation of corrective attachment relationships that allowed for the development of trust (Bennett, 2024). A study comparing adult CSA survivors with clinical vs. nonclinical levels of distress suggested that resolving traumatic attachment to the perpetrator and reclaiming trust were core therapeutic tasks that enabled expressing grief and anger and developing empowered meaning attributions (Leahy et al., 2003). Among survivors of intrafamilial CSA who had post-abuse contact with the parent, many believed that their contact experience had helped more than hindered their recovery, provided that the contact situation was physically safe. The potential for benefit was greatest where a mutual understanding was established (e.g., both agreed that the abuse did happen and that the parent was responsible), but long-term benefits, e.g., in fear reduction and knowing “where I stand” also occurred in the absence of mutual agreement (Paige and Thornton, 2015). In CSA survivors in recovery from substance abuse, self-change led to interpersonal insulation, which involved screening interactional situations in order to avoid dangerous persons and protect one’s self and children from harm from such persons, and locating and connecting with supportive, trustworthy persons (Hall, 2003). Sexual minority women and nonbinary individuals experienced healing from sexual trauma through interpersonal connection, seeking and receiving social support from others, and healing with others (Ellis and Wieling, 2024). Second generation Mexican American women survivors of sexual violence indicated that their communities at times posed barriers but nevertheless were important sources of support (Tummala-Narra et al., 2023).

Survivors of sexual abuse sought validation and support through disclosure in media and therapy. Women who had disclosed their sexual assault experience through various media channels experienced interpersonal support by having their accounts of the experience believed by others, unlike the criminal justice system for some survivors (Gueta et al., 2020). Adult female survivors of CSA experienced unique barriers and motivators over the course of their lives that resulted in seeking out and utilizing individual counseling, often due to circumstances not directly related to their CSA experiences. Survivors often did not initially conceptualize CSA as a necessary focus of treatment (Houseknecht, 2023).

### Theme 8: A secret (not) to be disclosed

Disclosure of sexual abuse contributed to meaning-making and recovery but carried risks of judgment and reidentification. Adult sexual abuse survivors experienced recovery through disclosing the abuse and making meaning of one’s trauma (Anderson and Hiersteiner, 2008). Women who have experienced sexual violence experienced telling their story as risky, an intersubjective encounter that must be managed and negotiated. In disclosing, they exposed themselves to reidentification, judgements and assumptions (Healicon, 2012). In women survivors of CSA, disclosure promoted a sense of empowerment, self-authority, having a choice, and wholeness as survivors no longer had to hide important aspects of themselves (Dodgson, 1996). Survivors’ coming-to-voice was influenced by their reasons for disclosing, e.g., survivors’ perceived importance of speaking out about the abuse history; risks survivors associated with disclosing; and survivors’ prior disclosure experiences during childhood, adulthood, and/or in therapy (Dodgson, 1996). In women CSA survivors seeking professional support, “going beyond themselves” explicated what survivors did with their story, who they told, and what they wanted to happen. Each woman had multiple contacts with persons or agencies that became a part of the overall experience of sexual assault (Murphy et al., 2009).

Themes in the social reactions that survivors received following disclosures of sexual violence were identified from unsolicited, de-identified personal correspondence in response to public testimony describing alleged sexual assault in a high-profile case (Brown et al., 2024). Positive reactions were appropriate action and emotional support in response to sexual violence disclosures; common negative reactions included dismissal, disbelief or denial, lack of repercussions for the perpetrator, retaliation, and victim blame. Survivors disclosing their sexual abuse online received helpful, harmful, and nuanced, mixed, and simultaneously harmful and helpful reactions (Driessen et al., 2024). A study of blog posts written by survivors of sexual assault found that bloggers frequently did not receive the supportive reaction to a face-to-face disclosure that they desired. Instead, blogging provided a safe environment that enabled victims to use their negative experience to educate and support others going through a similar experience, therefore allowing the bloggers to achieve some positive growth (Fawcett and Shrestha, 2016). Women who had disclosed their sexual assault experience through various media channels forged a resilient and activist identity, although public disclosure could also undermine their sense of security and make victimization a central feature of their identity (Gueta et al., 2020).

Clergy-perpetrated CSA survivors considered self-disclosure important in posttraumatic growth, combined with the social support that they received from the people closest to them after their disclosure (Sicilia et al., 2024). In young Indian female survivors of sexual violence, inability to share with the parents led to active suppression of the details of the incident, resulting in feelings of loneliness, but some recounted instances where they could confide in a specific family member (Fayaz et al., 2025). Young unmarried Indian female survivors of sexual violence used close female friendships combined with access to therapy to recognize and (re)frame experiences of sexual violence within an intimate relationship (Saxena and Oosterhoff, 2023). The discovery of self and a reconnection to culture and the ancestors encouraged African American women survivors of CSA to speak one’s truth into survival, and beyond (Stoner-Harris, 2024).

### Theme 9: Positive change

Adult survivors of sexual abuse gained awareness, rebuilt a sense of agency and identity, actively worked on their recovery, and reclaimed purpose in life, thereby experiencing growth and transformation. Adult CSA survivors referred to past, present, and future to describe and narrate their understanding of adjustments to their self-structure. A starting point of meaning-making was followed by a stage of gaining new awareness, leading to an adjusted position, suggesting a movement and fluidity to their recovery (Wright and Gabriel, 2018). In female CSA survivors, important sources of active healing included therapy, (in)formal education, compassion and empathy, blame attribution to abusers, and confronting abusers (Arias and Johnson, 2013). Adult survivors of incest, after grappling with and figuring out the meaning of the CSA, tackled the effects of the CSA, and laid claim to their life – living an empowered life (Draucker et al., 2011). Sexual abuse survivors replaced long-standing feelings of powerlessness with a new sense of agency, reclaiming a positive identity from a “damaged” self-definition (Harvey et al., 2000), changing destructive to constructive rumination (Walker-Williams and Fouché, 2018). Women survivors of adult sexual assault engaged in a process of rebuilding the self through accepting, connecting, reclaiming power, and creating meaning and purpose, which fostered personal growth and transformation (Olson, 2015). Most women CSA survivors perceived at least some benefit resulting from coping with the CSA experience, e.g., religious or spiritual growth, improved relationships with others, and improved parenting skills (Wright et al., 2007).

Survivors of sexual abuse developed their relationship to themselves, others, and the world, their appreciation for life, a survivor identity, day-to-day practices of care and connection, and self-centering. Some rape survivors noted that recovering from the trauma made them appreciate life more. “I remind myself every day of how valuable this day is. .. I have a reason to be here” (Smith and Kelly, 2001, p. 346). Female CSA survivors who focused on the positive things in themselves, in their life, and on seeking meaning in the abuse felt able to relate to life better after the trauma, feeling they had become better people or better parents, and understanding the family or social dynamics surrounding the abuse (Wright et al., 2007). Military sexual trauma survivors described altering their attitude toward unavoidable suffering by developing a survivor mentality code, a view of self in the world, and resiliency (Preston et al., 2023). For female undergraduate survivors of campus unwanted sexual experiences, identified healing goals included cultivating worthiness, and day-to-day healing moments of self-care, self-love, connection, hope, peace, and freedom contributed to achieving these (Sinko, 2019). In CSA survivors recovering from substance abuse, areas of self-change included self-centering (i.e., naming and focusing on one’s own life and interests, trusting one’s own instincts and perceptions), ownership, interpersonal insulation, willfulness, seeing options, and spiritual connection. They considered all of these positive transitions they made after adversity, especially abuse and its consequences (Hall, 2003).

### Theme 10: Activism

Adult sexual assault survivors who were engaged in anti–sexual assault activism described that activism helped them find their voice and regain their power, moving from silence and shame around their sexual assault to freedom and empowerment. Their involvement in activism helped increase their understanding of themselves and their sexual assault experience, served as a useful coping mechanism, improved their self-confidence and relationships, allowed them to stand up and speak out against attitudes and behaviors that foster rape culture, made them feel supported, validated, and connected to others, and provided a source of meaning and fulfillment in their lives (Strauss Swanson and Szymanski, 2020). However, challenges associated with anti–sexual assault activism included being triggered, being inundated with media coverage and public narratives about sexual assault, burning out, and feeling disillusioned and frustrated (Strauss Swanson and Szymanski, 2020). Adult activists, who were also CSA survivors, perceived anti-sexual assault activism as transforming their lives by empowering them, challenging injustice, and advocating for systemic change, emphasizing activism’s dual role of personal healing and societal impact (Attrash-Najjar and Katz, 2025). In adult survivors of incest who had experienced some degree of healing, “constructing a personal residence” involved influencing the community in a meaningful way (Draucker, 1992). Recognizing CSA as a social phenomenon reflected new understandings about their personal abuse histories in the context of a broader social problem. With this awareness, many survivors became involved in social activism (Dodgson, 1996). Willfulness in CSA survivors recovering from substance abuse implied defining one’s path in life, relying on one’s self for action, holding strong opinions, and having an impact or causing change in the environment (Hall, 2003).

Women who had disclosed their sexual assault experience through various media channels were motivated by a desire to advocate for social change and to find meaning, thereby facilitating their own recovery. They saw as a healing experience because it helped them forge a resilient and activist identity (Gueta et al., 2020). Sexual minority women and nonbinary individuals politicized their posttraumatic growth and understood their struggle as connected to larger systems of oppression, experiencing iteratively individual and relational process of queer worldmaking (Ellis and Wieling, 2024).

### Theme 11: Honoring strengths and hope

Survivors of sexual abuse relied on self-support and stood up for themselves amid systemic and relational challenges and derived hope from images of the achievable future. Women who had been sexually abused as children indicated that the recovery process made them emotionally stronger and more capable of handling problems than they would have been otherwise; they saw themselves and their future as getting better in the years ahead (Bogar and Hulse-Killacky, 2006). Clergy-perpetrated CSA survivors retained a sense of hope: “What else can I lose? If I have lost everything. .. I live day by day, and I only hope that what is coming will be good” (Sicilia et al., 2024). Standing up for oneself and supporting themselves remained the only option for CSA survivors in India, a patriarchal society, if external support was not readily available (Fayaz et al., 2025). In CSA survivors in recovery from substance abuse, *epiphanies* were experiences that broke routines and revealed negative dynamics that had become routine, providing an image of what life could be like, of the achievable future (Hall, 2003). Maintaining momentum between epiphanies involved processes that sustain positive self-change (Hall, 2003).

Survivors, particularly those from marginalized communities, reclaimed themselves through culturally grounded aspirations for collective healing and future empowerment. Diverse culturally grounded, sociopolitical trauma-informed pathways for survivors to reclaim themselves, especially for marginalized survivors, included culture as medicine (traditional cultural healers), community support, spirituality and religiosity, expressive arts, and resistance (Bryant, 2024). Black LGBTQ + girls, femmes, transgender and nonbinary youth and young adults, while working to actively set and pursue goals around educational attainment and career advancement, articulated the desire for themselves and their communities to live free lives, deriving strength from aspirations and dreams for their futures and the futures of their communities (Mountz et al., 2024). Black young women sexual violence survivors expressed a community orientation in understanding both the harm and healing of cultural betrayal trauma and provided recommendations for community-level solidarity and healing (Gomez and Gobin, 2024). In eastern Democratic Republic of Congo, women survivors of war-related rape faced challenges, such as isolation, feelings of shame, interpersonal difficulties, and hesitancy to seek medical and psychological treatment, stemming from their communities’ interpretation of rape as a sexual taboo and as a danger to the survivors’ families and communities. Survivors indicated that person-focused values have the potential to rally the community in solidarity and support for survivors (Maisha et al., 2024). For Maori survivors of familial CSA, spirituality, care and reciprocity, collectivity and unity acted as sources of resilience; access to practices keeping with the love, care, commitment and respect that reflected ancestral principles created long-lasting change in their family unit, providing protection of future generations (Harrison and Le Grice, 2023).

### Theme 12: Living with triggers

Adult survivors of childhood sexual abuse faced ongoing challenges in recovery, as triggers from life events, continued contact with perpetrators, and experiences like pain revived traumatic memories and vulnerabilities. In adult sexual abuse survivors, managing memories constituted one domain of healing (Draucker et al., 2009). Some adult survivors of CSA were hindered in their recovery because the perpetrator was still in their close environment: “... at the time my abuser was still in contact with me at family gatherings and different things and he came and visited one day. .. and it felt just like l was back to eight years old” (Chouliara et al., 2014, p. 73). Life events acted as triggers that brought back memories and feelings about CSA, prompting survivors to go back and trace earlier steps of their recovery process: “I think that there would always be a slightly vulnerable part of me. A part of me that might be more easily wounded in a situation than maybe someone else. A part of me. .. that does still get scared. .. I suppose I say to me, look I do have a vulnerable part of me and it’s important that I constantly be aware of that” (Chouliara et al., 2014, p. 75). Survivors of sexual trauma receiving a tattoo described how pain prompted triggering memories: “When you spark, when you hit that pain receptor, it clicks that image that you remember.. .” (Maxwell et al., 2024, p. 450). CSA survivors in recovery from substance abuse described that basic and developmental issues were carried into new negative experiences: “Abuse is the stem and root of all that has happened in my life” (Hall, 2000, p. 621).

Child sexual abuse survivors navigated complex processes of reclaiming their sexuality and rebuilding intimate relationships. In CSA survivors, *determining my sexuality* involved a process to understand what the abuse was (e.g., was it rape? love?), why it happened (e.g., was it my fault?), and what it did to their sexuality (did it make me promiscuous?). *Laying claim to one’s sexuality* referred to the process of asserting one’s right to one’s own sexuality (Draucker et al., 2011). Young unmarried Indian female victim-survivors of sexual violence used their close personal and therapeutic networks to understand how to build more satisfying intimate relations. This sometimes involved meeting with the ex-partner to discuss the abuse (Saxena and Oosterhoff, 2023). In African American women survivors, reframing their experiences of CSA involved externalizing racist and sexist stereotypes of African American women and reclaiming sexuality (Singh et al., 2013). Women CSA survivors living in the metropolitan area of Mexico City who took part in therapy, attributed various meanings to the sexual “effects” of CSA, specifically, losing and recovering sexual desire, fighting off sex fear, harm trying to prevail, and “going out alive after a nightmare”, rediscovering sexual wellbeing (Guzmán-Díaz and Trujano Ruiz, 2023). Among survivors of childhood psychological maltreatment and CSA, sexuality and intimate relationships were often poor, but opportunities to develop mindfulness dispositions appeared to enhance posttraumatic growth in survivors’ intimate relationships (Dussault et al., 2024). Women who had become involved in commercial sexual exploitation after having survived CSA, experienced internal compulsions that arose because of this abuse (Dean, 2022): “When it was happening, it felt like I was being promiscuous, like I was seeking it. Because I felt like it worked. It was the first thing in my life that got people to pay attention to me, that made people value me or care about me (Dean, 2022, p. 194).”

Child sexual abuse survivors who became parents often broke the cycle of abuse and fostered generativity by mentoring and safeguarding others. *Living the family legacy* involved passing on the abuse, wishing and attempting to stop and actually stopping the abuse cycle (Draucker et al., 2011). Thriving adult female CSA survivors displayed remarkable generativity, parenting their own children well, assuring they were never in unsafe circumstances, and mentoring other young victims of abuse (Thomas and Hall, 2008).

### Overarching themes

**Figure 2:**
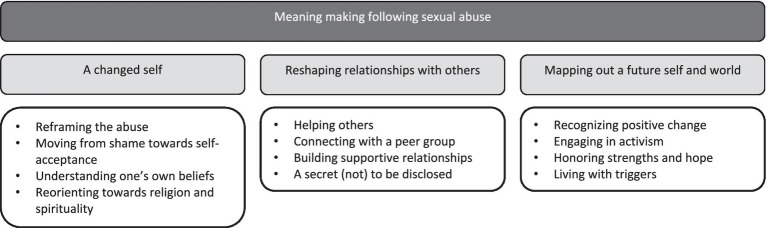
Themes and overarching themes.

We grouped the 12 themes into three overarching themes consisting of each four themes: a changed self, reshaping connections with others, and mapping out a future self and world. Figure 2 illustrates the grouping of the themes. In the overarching theme a changed self, themes included: reframing the abuse, moving from shame towards self-acceptance, understanding one’s own beliefs, and reorienting toward spirituality and religion. In the overarching theme reshaping relationships with others, themes included: helping others, connecting with a peer group, building supportive relationships, and a secret (not) to be disclosed. In the overarching theme envisioning a future self and world, themes included: recognizing positive change, engaging in activism, honoring strengths and hope, and living with triggers.

## Discussion

This meta-synthesis of qualitative studies exploring meaning-making in survivors following sexual abuse found 12 themes that reflect how survivors attribute meaning to their experiences of sexual abuse. The process of meaning-making unfolds over time mostly subconsciously and involves turning points and transitions, e.g., from receiving to providing support, from retelling to reframing, and deliberate acts of self-care. Survivors’ perceptions of the role of meaning-making in recovery highlights the beneficial potential of meaning-making, suggesting that the process of meaning-making is adaptive and results in sense-making, benefit finding, and identity (Gillies and Neimeyer, 2006). Our findings align with a theoretical framework that distinguishes between global and situational meaning (Park, 2010; Park et al., 2012; Park and Ai, 2006). The attribution of meaning involves a negotiation between situational meanings attributed – e.g., to having been exposed to sexual abuse – and global meanings (i.e., meaning-in-life).

Meaning-making involves connecting experiences on different levels, including existential, cognitive, affective, motivational, social, and bodily levels (Smid et al., 2026). Existential orientations constitute “how we find ourselves in a world” (Ratcliffe, 2008, p. 2) and provide us with a sense of reality: of ourselves, our actions, other persons and objects, and the surrounding world as such (Stephan, 2012). Disorientation is an inherent part of existential meaning-making. Disorienting experiences foreground the question of what ‘ultimately’ matters to us and how to orient towards corresponding visions of the good (Schuhmann et al., 2026). Importantly, mental healthcare providers aiming to support meaning-making may identify therapeutic goals across levels of meaning, e.g., identifying values (existential), constructing a story (cognitive), acceptance (affective), active engagement with the future (motivational), reengaging with others (social) and self-care (bodily).

**Figure 3:**
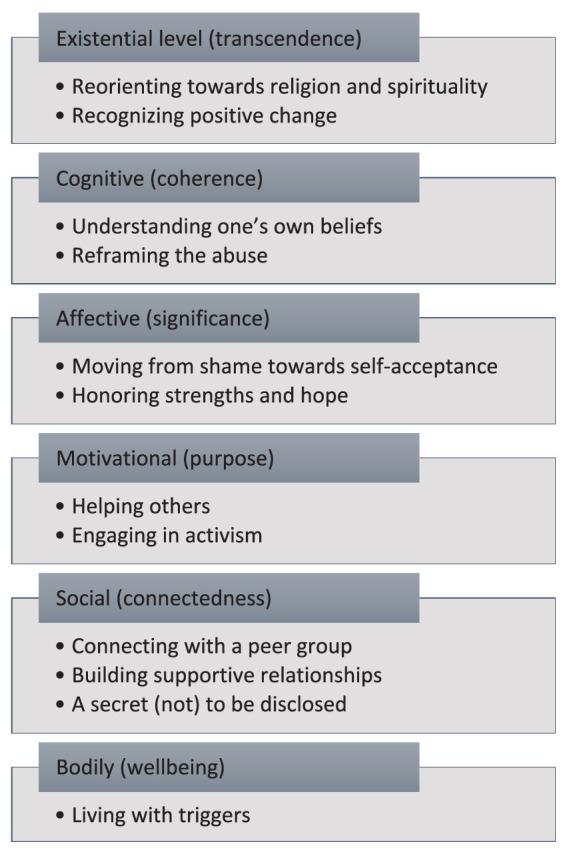
Meaning-making following sexual abuse across levels of meaning.

The findings from this meta-synthesis suggest that meaning-making following sexual abuse likewise occurs on different levels, as summarized in Figure 3. Existentially, meaning-in-life involves feeling connected to something larger than oneself, a sense of belonging to a meaningful whole that transcends individual existence. This transcendence, the human capacity to transform both self and world, is rooted in the belief that change, creativity, and the growth of meaning are possible (Johnson, 2012). Our findings suggest that meaning-making following sexual abuse on the existential level involves reorienting towards spirituality and religion and recognizing positive change. Cognitively, affectively, and motivationally, meaning-in-life is characterized by coherence (a sense of comprehensibility), significance (a feeling that life is important and valuable), and purpose (having core goals and direction) (Martela and Steger, 2016). Our findings suggest that meaning-making following sexual abuse on the cognitive level involves understanding one’s own beliefs and reframing the abuse; on the affective level, it involves moving from shame towards self-acceptance and honoring strengths and hope; and on the motivational level, it involves helping others and engaging in activism.

Socially, meaning-in-life is linked to social connectedness, which bolsters community resilience after trauma and loss (Hobfoll et al., 2007; Stavrova and Luhmann, 2016). On a bodily level, meaning-in-life is vital for physical well-being, health, and quality of life (Haugan and Dezutter, 2021), and a meta-analysis has even demonstrated a link between physical health and meaning-related feelings of harmony, peace, and well-being (Czekierda et al., 2017). Our findings suggest that meaning-making following sexual abuse on the social level involves connecting with a peer group, building supportive relationships and a secret (not) to be disclosed; on the bodily level, it involves living with triggers.

Meaning-making following sexual abuse on a social level may be enhanced by societal responses to sexual abuse, which include different forms of justice and compensation. A systematic review identified numerous barriers to reporting, investigating, prosecuting, and sentencing of cases of adult sexual assault in the criminal justice system, emphasizing an urgent need for reform of the criminal justice system’s response to sexual assault and rape (Wieberneit et al., 2024). A scoping review on the experiences and perceptions of sexually abused children as participants in the legal process emphasized children’s need for validation and their related needs to be protected, seen and heard, believed, and provided with support, arguing for a holistic approach with children throughout the legal process (Field and Katz, 2023). In a study of the impact of compensation on CSA survivors’ mental wellbeing, survivors emphasized the importance of the validation that a crime had been committed against a child (Briggs et al., 2023). In addition, financial assistance enabled them to gain access to intervention and support services.

### Study strengths and limitations

Study strengths include the large number of studies with a broad representation of survivors of sexual abuse – both CSA and adult sexual assault – across studies, from various abuse and post-abuse contexts as well as cultural contexts. The focus on qualitative studies aligns with the subjective and unique nature of meaning-making experiences that are best understood through qualitative methods. Across the included studies, considerable multiplicity in methodological approaches, participant characteristics, and contextual factors emerged. In line with the quality practice of reflexive thematic analysis, we understood meaning and knowledge as situated and contextual, and we therefore believe this multiplicity can enhance the transferability of the findings (Braun and Clarke, 2021, 2024).

Several limitations need to be acknowledged. These include the methodological limitations of the included studies, such as the paucity of longitudinal studies. Included studies are predominantly cross-sectional, relying on participants’ memory of their meaning-making experiences. We aimed to also include mixed methods studies, specifically studies integrating quantitative and qualitative research questions, methods, and findings (Matović and Ovesni, 2023); however, during our systematic search, no such study was identified. Limitations of the current review include the search and selection of studies. Included studies were only in English language and predominantly from the US, potentially limiting the transferability of the findings. Nevertheless, the studies covered considerable cultural heterogeneity. Due to variations in wordings, studies that our systematic search strategy failed to identify might have represented additional perspectives.

Directions for future research

Future research should investigate variations in meaning-making following sexual abuse based on factors likely to be relevant, such as the pervasiveness and severity of the abuse, whether the abuse was chronic or a single incident, the survivor’s relationship to the perpetrator, as well as gender and age. Such variation can be examined through qualitative approaches, including the constant comparative method (Boeije, 2002). This method involves iterative categorizing, coding, refining, and linking categories, which goes hand in hand with theoretical sampling. Researchers determine what data to collect next – and from whom – based on provisional theoretical insights, then compare newly gathered data with existing data to deepen the analysis.

Variations in meaning-making are also likely to emerge within underrepresented groups, including male survivors and survivors with diverse sexual orientations, gender identities, and gender expressions. Clarifying meaning-making processes among male survivors, in particular, may contribute to preventing the intergenerational transmission of abuse. Additionally, future studies are needed to explore the impact of societal validation, through mechanisms such as justice and compensation, on survivors’ meaning-making and recovery.

There is a pressing need for studies involving young people in the period shortly after abuse or rape has occurred. In addition to biographical narratives (e.g., Krayer et al., 2015; Winterstein et al., 2023) and life-span perspectives (e.g., Draucker et al., 2011), longitudinal research using repeated interviews (e.g., Duma et al., 2007a, 2007b) is essential for developing developmental models that can better inform clinicians working with young people.

### Implications for practice

Therapeutic models need to incorporate the role of meaning-making in the recovery of survivors of sexual abuse. Meaning-making on different levels – existential, cognitive, affective, motivational, social, and bodily levels – can guide interdisciplinary collaboration and deepen professionals’ understandings of their roles and responsibilities within trauma-informed care (Raja et al., 2015). Recognizing the potential of collective healing is essential, as many therapeutic approaches are predominantly individual in focus. Strengthening peer and community support, fostering cultural connectedness, and promoting social change can contribute to preventing sexual abuse and reducing its long-term impacts.

## Data Availability

The original contributions presented in the study are included in the article/Supplementary material, further inquiries can be directed to the corresponding author/s.

## References

[ref1] Anderson K. M. Hiersteiner C. (2008). Recovering from childhood sexual abuse: is a “storybook ending” possible? Am. J. Fam. Ther. 36, 413–424. doi: 10.1080/01926180701804592

[ref2] Arias B. J. Johnson C. V. (2013). Voices of healing and recovery from childhood sexual abuse treatment of childhood sexual abuse survivors. J. Child. Sex. Abuse 22, 822–841. doi: 10.1080/10538712.2013.830669 24125084

[ref3] Attrash-Najjar A. Katz C. (2025). Meanings of anti-sexual assault activism among adults who underwent child sexual abuse: “I survived by knowing that I was going to act.”. Child Abuse Negl. 161:107249. doi: 10.1016/j.chiabu.2025.107249, 39837170

[ref4] Bainbridge A. (2022). Exploring self-disclosure between the survivor-therapist and survivor-clients: an autoethnography of the value of ‘sisterhood’ between female survivors of sexual violence. Couns. Psychother. Res. 22, 1087–1099. doi: 10.1002/capr.12573

[ref5] Barker C. Ford S. Eglinton R. Quail S. Taggart D. (2023). The truth project paper one—how did victims and survivors experience participation? Addressing epistemic relational inequality in the field of child sexual abuse. Front. Psych. 14:1128451. doi: 10.3389/fpsyt.2023.1128451, 37333914 PMC10272443

[ref6] Bennett K. (2024). Relational trust in adult survivors of childhood sexual abuse: a grounded theory study. Dissertation Abstracts International Section A: Humanities and Social Sciences. ProQuest Dissertations & Theses, Capella University

[ref7] Beveridge K. Cheung M. (2004). A spiritual framework in incest survivors treatment. J. Child Sex. Abus. 13, 105–120. doi: 10.1300/J070v13n02_06, 15388414

[ref8] Boeije H. (2002). A purposeful approach to the constant comparative method in the analysis of qualitative interviews. Qual. Quant. 36, 391–409. doi: 10.1023/A:1020909529486

[ref9] Bogar C. B. Hulse-Killacky D. (2006). Resiliency determinants and resiliency processes among female adult survivors of childhood sexual abuse. J. Couns. Dev. 84, 318–327. doi: 10.1002/j.1556-6678.2006.tb00411.x

[ref10] Bonanno G. A. (2004). Loss, trauma, and human resilience: have we underestimated the human capacity to thrive after extremely aversive events? Am. Psychol. 59, 20–28. doi: 10.1037/0003-066X.59.1.20, 14736317

[ref11] Bonanno G. A. (2013). Meaning making, adversity, and regulatory flexibility. Memory 21, 150–156. doi: 10.1080/09658211.2012.745572, 23311413 PMC3565080

[ref12] Bramer W. M. Bain P. (2017). Updating search strategies for systematic reviews using EndNote. J. Med. Libr. Assoc. 105, 285–289. doi: 10.5195/jmla.2017.183, 28670219 PMC5490709

[ref13] Bramer W. M. Giustini D. De Jonge G. B. Holland L. Bekhuis T. (2016). De-duplication of database search results for systematic reviews in EndNote. J. Med. Libr. Assoc. 104, 240–243. doi: 10.5195/jmla.2016.24, 27366130 PMC4915647

[ref14] Brandstätter M. Baumann U. Borasio G. D. Fegg M. J. (2012). Systematic review of meaning in life assessment instruments. Psychooncology 21, 1034–1052. doi: 10.1002/pon.2113, 22232017

[ref15] Braun V. Clarke V. (2006). Using thematic analysis in psychology. Qual. Res. Psychol. 3, 77–101. doi: 10.1191/1478088706qp063oa

[ref16] Braun V. Clarke V. (2021). One size fits all? What counts as quality practice in (reflexive) thematic analysis? Qual. Res. Psychol. 18, 328–352. doi: 10.1080/14780887.2020.1769238

[ref17] Braun V. Clarke V. (2024). Supporting best practice in reflexive thematic analysis reporting in palliative medicine: a review of published research and introduction to the reflexive thematic analysis reporting guidelines (RTARG). Palliat. Med. 38, 608–616. doi: 10.1177/02692163241234800, 38469804 PMC11157981

[ref18] Briggs L. O’Leary P. Grove J. (2023). The impact of critical life points and events for survivors of institutional child sexual abuse: understanding trauma and the role of compensation in recovery. J. Interpers. Violence 38, 2742–2758. doi: 10.1177/08862605221102480, 35617673

[ref19] Brown T. Michael E. Felber E. Gore-Felton C. Ford C. B. (2024). Narratives on disclosure of sexual violence: survivors’ responses to public testimony to a high-profile case. Psychol. Trauma Theory Res. Pract. Policy. WoS LB:39418408. doi: 10.1037/tra0001778 39418408

[ref20] Bryant T. (2024). Lessons from decolonial and liberation psychologies for the field of trauma psychology. Am. Psychol. 79, 683–696. doi: 10.1037/amp0001393, 39172373

[ref21] Bryant-Davis T. Wong E. C. (2013). Faith to move mountains: religious coping, spirituality, and interpersonal trauma recovery. Am. Psychol. 68, 675–684. doi: 10.1037/a0034380, 24320650

[ref22] Carey L. B. Hodgson T. J. (2018). Chaplaincy, spiritual care and moral injury: considerations regarding screening and treatment. Front. Psych. 9:619. doi: 10.3389/fpsyt.2018.00619, 30568605 PMC6290645

[ref23] Carroll C. Booth A. (2015). Quality assessment of qualitative evidence for systematic review and synthesis: is it meaningful, and if so, how should it be performed? Res. Synth. Methods 6, 149–154. doi: 10.1002/jrsm.1128, 26099483

[ref24] Chouliara Z. Karatzias T. Gullone A. (2014). Recovering from childhood sexual abuse: a theoretical framework for practice and research. J. Psychiatr. Ment. Health Nurs. 21, 69–78. doi: 10.1111/jpm.12048, 23379783

[ref25] Czekierda K. Banik A. Park C. L. Luszczynska A. (2017). Meaning in life and physical health: systematic review and meta-analysis. Health Psychol. Rev. 11, 387–418. doi: 10.1080/17437199.2017.1327325, 28488471

[ref26] Dean J. (2022) Child sexual abuse and the link to commercial sexual exploitation [Ph.D., Institute for Clinical Social Work (Chicago)]. In ProQuest Dissertations and Theses. Available online at: https://www.proquest.com/docview/2688950955/abstract/CA8844E1EFFB4789PQ/1?sourcetype=Dissertations%20&%20Theses

[ref28] Dodgson J. (1996) A qualitative exploration of women survivors’ disclosure of childhood sexual abuse. Dissertations. Available online at: https://scholarworks.wmich.edu/dissertations/1691 [Accessed November 7, 2024].

[ref29] Draucker C. B. (1992). The healing process of female adult incest survivors: constructing a personal residence. Image J. Nurs. Scholarsh. 24, 4–8. doi: 10.1111/j.1547-5069.1992.tb00691.x, 1541470

[ref30] Draucker C. B. Martsolf D. S. Roller C. Knapik G. Ross R. Stidham A. W. (2011). Healing from childhood sexual abuse: a theoretical model. J. Child Sex. Abus. 20, 435–466. doi: 10.1080/10538712.2011.588188, 21812546 PMC3970162

[ref31] Draucker C. B. Martsolf D. S. Ross R. Cook C. B. Stidham A. W. Mweemba P. (2009). The essence of healing from sexual violence: a qualitative metasynthesis. Res. Nurs. Health 32, 366–378. doi: 10.1002/nur.20333, 19415681 PMC3235413

[ref32] Driessen M. C. Bhuptani P. H. Kiefer R. Peterson R. Mayer E. Cruz-Sanchez M. . (2024). Reactions to and impact of survivor online disclosures: a qualitative analysis. J. Child Sex. Abuse 33, 951–969. doi: 10.1080/10538712.2024.2428287, 39530536 PMC12010705

[ref33] Duma S. E. Mekwa J. N. Denny L. D. (2007a). Women’s journey of recovery from sexual assault trauma: a grounded theory - part 1. Curationis 30, 4–11. doi: 10.4102/curationis.v30i4.1111, 18402415

[ref34] Duma S. E. Mekwa J. N. Denny L. D. (2007b). Women’s journey of recovery from sexual assault trauma: a grounded theory - part 2. Curationis 30, 12–20. doi: 10.4102/curationis.v30i4.1112, 18402416

[ref35] Dussault E. Fernet M. Guyon R. Godbout N. (2024). Mindfulness and posttraumatic growth in childhood sexual abuse and psychological maltreatment survivors. Can. J. Hum. Sex. 33, 72–85. doi: 10.3138/cjhs-2023-0006

[ref36] Dworkin E. R. Menon S. V. Bystrynski J. Allen N. E. (2017). Sexual assault victimization and psychopathology: a review and meta-analysis. Clin. Psychol. Rev. 56, 65–81. doi: 10.1016/j.cpr.2017.06.002, 28689071 PMC5576571

[ref37] Ellis E. Wieling E. (2024). Not just growth, but worldmaking: a phenomenological exploration of posttraumatic growth among sexual minority women and nonbinary individuals. Am. Psychol. 79, 1202–1213. (39531716). doi: 10.1037/amp0001332, 39531716

[ref38] Fawcett H. Shrestha L. (2016). Blogging about sexual assault: a thematic analysis. J. Forensic Pract. 18, 39–51. doi: 10.1108/JFP-05-2015-0032

[ref39] Fayaz I. Rizvi M. Gupta I. (2025). Strength in adversity: a qualitative study on resilience among Indian survivors of sexual violence. J. Evid. Based Soc. Work 22, 448–468. doi: 10.1080/26408066.2025.2467903, 39972527

[ref40] Field N. Katz C. (2023). The experiences and perceptions of sexually abused children as participants in the legal process: key conclusions from a scoping literature review. Trauma Violence Abuse 24, 2758–2771. doi: 10.1177/15248380221111463, 35762223

[ref41] Gillies J. M. Neimeyer R. A. (2006). Loss, grief, and the search for significance: toward a model of meaning reconstruction in bereavement. J. Constr. Psychol. 19, 31–65. doi: 10.1080/10720530500311182

[ref42] Gomez J. M. Gobin R. L. (2024). “It will always feel worse because it comes with that added ‘betrayal’”: intersectionality praxis and black young women survivors’ perspectives on cultural betrayal trauma theory. J. Trauma Dissociation 25, 656–673. doi: 10.1080/15299732.2024.2383197, 39093694

[ref43] Gueta K. Eytan S. Yakimov P. (2020). Between healing and revictimization: the experience of public self-disclosure of sexual assault and its perceived effect on recovery. Psychol. Violence 10, 626–637. doi: 10.1037/vio0000309

[ref44] Guzmán-Díaz A. V. Trujano Ruiz P. (2023). Co-constructing women’s stories about the sexual effects of child sexual abuse: a relational constructionist approach. Qual. Psychol. 10, 521–532. doi: 10.1037/qup0000251

[ref45] Hailes H. P. Yu R. Danese A. Fazel S. (2019). Long-term outcomes of childhood sexual abuse: an umbrella review. Lancet Psychiatry 6, 830–839. doi: 10.1016/S2215-0366(19)30286-X, 31519507 PMC7015702

[ref46] Hall J. M. (2000). Core issues for female child abuse survivors in recovery from substance misuse. Qual. Health Res. 10, 612–631. doi: 10.1177/104973230001000504, 11066868

[ref47] Hall J. M. (2003). Positive self-transitions in women child abuse survivors. Issues Ment. Health Nurs. 24, 647–666. doi: 10.1080/01612840305325, 12907381

[ref48] Harrison N. Le Grice J. (2023). “Family relatedness for Maori survivors of familial childhood sexual abuse,” in Relationships and Mental Health: Relational Experience in Distress and Recovery, eds Z. Boden-Stuart & M. Larkin (Cham: Palgrave Macmillan), 61–80. doi: 10.1007/978-3-031-50047-3_4

[ref49] Harvey M. R. Mishler E. G. Koenen K. Harney P. A. (2000). In the aftermath of sexual abuse: making and remaking meaning in narratives of trauma and recovery. Narrat. Inq. 10, 291–311. doi: 10.1075/ni.10.2.02har

[ref50] Haugan G. Dezutter J. (2021). “Meaning-in-life: a vital salutogenic resource for health,” in Health Promotion in Health Care – Vital Theories and Research, eds. Haugan G. Eriksson M. (Cham: Springer International Publishing), 85–101. 36315724

[ref51] Healicon A. (2012). Telling the truth: using narrative accounts of sexual violence to trouble feminist and therapeutic theory. Power Educ. 4, 33–44. doi: 10.2304/power.2012.4.1.33

[ref52] Hegarty S. Ehntholt K. Williams D. Kennerley H. Billings J. Bloomfield M. (2022). Acceptability and mechanisms of change associated with group cognitive behavioural therapy using the recovering from childhood abuse Programme among women with CPTSD: a qualitative analysis. Cogn. Behav. Ther. 15:e46. doi: 10.1017/S1754470X2200037X

[ref53] Herman J. L. (1992). Trauma and Recovery. New York: Basic Books Inc., 1992.

[ref54] Hobfoll S. E. Watson P. Bell C. C. Bryant R. A. Brymer M. J. Friedman M. J. . (2007). Five essential elements of immediate and mid-term mass trauma intervention: empirical evidence. Psychiatry 70, 283–315. doi: 10.1521/psyc.2007.70.4.283 18181708

[ref55] Houg B. L. (2008). The role of spirituality in the ongoing recovery process of female sexual abuse survivors. University Digital Conservancy. Available online at: https://hdl.handle.net/11299/46810 [Accessed November 7, 2024].

[ref56] Houseknecht A. (2023). A narrative inquiry study of adult female survivors of childhood sexual abuse and their journey into individual counseling. Available online at: https://scholarworks.wmich.edu/dissertations/3962 [Accessed November 7, 2024].

[ref57] Johnson M. (2012). The Meaning of the body: Aesthetics of human Understanding. Chicago: University of Chicago Press.

[ref58] Knapik G. P. Martsolf D. S. Draucker C. B. (2008). Being delivered: spirituality in survivors of sexual violence. Issues Ment. Health Nurs. 29, 335–350. doi: 10.1080/01612840801904274, 18382913 PMC3155866

[ref59] Krayer A. Seddon D. Robinson C. A. Gwilym H. (2015). The influence of child sexual abuse on the self from adult narrative perspectives. J. Child Sex. Abus. 24, 135–151. doi: 10.1080/10538712.2015.1001473, 25747417

[ref60] Krug E. G. Dahlberg L. L. Mercy J. A. Zwi A. B. Lozano R. (2002). World Report on Violence and Health. Geneva: World Health Organization.

[ref61] Leahy T. Pretty G. Tenenbaum G. (2003). Childhood sexual abuse narratives in clinically and nonclinically distressed adult survivors. Prof. Psychol. Res. Pract. 34, 657–665. doi: 10.1037/0735-7028.34.6.657

[ref62] Lebowitz L. Roth S. (1994). “I felt like a slut”: the cultural context and women’s response to being raped. J. Trauma. Stress. 7, 363–390. doi: 10.1002/jts.2490070304, 8087400

[ref63] Liu H. Petukhova M. V. Sampson N. A. Aguilar-Gaxiola S. Alonso J. Andrade L. H. . (2017). Association of DSM-IV posttraumatic stress disorder with traumatic experience type and history in the World Health Organization world mental health surveys. JAMA Psychiatry 74, 270–281. doi: 10.1001/jamapsychiatry.2016.3783, 28055082 PMC5441566

[ref64] Maisha B. Anglin J. Mwindo T. Tomsine C. Florent S. M. (2024). Exploring cultural factors in the “systemic revictimization” of rape survivors in Mwenga (DRC). Soc. Sci. 13:411. doi: 10.3390/socsci13080411

[ref65] Martela F. Steger M. F. (2016). The three meanings of meaning in life: distinguishing coherence, purpose, and significance. J. Posit. Psychol. 11, 531–545. doi: 10.1080/17439760.2015.1137623

[ref66] Matović N. Ovesni K. (2023). Interaction of quantitative and qualitative methodology in mixed methods research: integration and/or combination. Int. J. Soc. Res. Methodol. 26, 51–65. doi: 10.1080/13645579.2021.1964857

[ref67] Maxwell D. Leat S. R. Thomas J. Thomas S. A. Coad S. (2024). The tattoo environment as a therapeutic healing space for sexual assault survivors. Deviant Behav. 45, 438–455. doi: 10.1080/01639625.2023.2250052

[ref68] Methley A. M. Campbell S. Chew-Graham C. McNally R. Cheraghi-Sohi S. (2014). PICO, PICOS and SPIDER: a comparison study of specificity and sensitivity in three search tools for qualitative systematic reviews. BMC Health Serv. Res. 14:579. doi: 10.1186/s12913-014-0579-0, 25413154 PMC4310146

[ref69] Millar G. M. Stermac L. (2000). Substance abuse and childhood maltreatment: conceptualizing the recovery process. J. Subst. Abus. Treat. 19, 175–182. doi: 10.1016/S0740-5472(00)00117-3, 10963929

[ref70] Mountz S. Dill L. J. Willows M. Dyett J. (2024). It’s okay to dream: navigating trauma, healing, and futuring among LGBTQ + black girls, transgender and nonbinary youth in New York state. Child Youth Serv. Rev. 163, 1–12. doi: 10.1016/j.childyouth.2024.107755

[ref71] Mughal F. B. Sinko L. Saint Arnault D. (2024). Enhancing the recovery process for undergraduate women survivors of sexual violence: identifying facilitators to healing. Violence Against Women 32, 70–87. doi: 10.1177/10778012241307334, 39703122

[ref72] Murphy S. B. Moynihan M. M. Banyard V. L. (2009). Moving within the spiral: the process of surviving. Affilia 24, 152–164. doi: 10.1177/0886109909331702

[ref73] Olson N. S. (2015). Positive adaptation in women following sexual assault: a grounded theory study—Washington State University. Available online at: https://hdl.handle.net/2376/6153 and https://rex.libraries.wsu.edu/esploro/outputs/doctoral/Positive-Adaptation-in-Women-Following-Sexual/99900581731001842 [Accessed November 7, 2024].

[ref74] Ouzzani M. Hammady H. Fedorowicz Z. Elmagarmid A. (2016). Rayyan—a web and mobile app for systematic reviews. Syst. Rev. 5:210. doi: 10.1186/s13643-016-0384-4, 27919275 PMC5139140

[ref75] Page M. J. McKenzie J. E. Bossuyt P. M. Boutron I. Hoffmann T. C. Mulrow C. D. . (2021). The PRISMA 2020 statement: an updated guideline for reporting systematic reviews. BMJ 372:n71. doi: 10.1136/bmj.n71 33782057 PMC8005924

[ref76] Paige J. Thornton J. (2015). Healing from intrafamilial child sexual abuse: the role of relational processes between survivor and offender. Child. Aust. 40, 242–259. doi: 10.1017/cha.2015.21

[ref77] Panepinto A. R. (2004) Meaning Reconstruction and Recovery in Rape Survivors [Miami University] Available online at: https://etd.ohiolink.edu/acprod/odb_etd/etd/r/1501/10?clear=10&p10_accession_num=miami1102005366 [Accessed November 7, 2024].

[ref78] Park C. L. (2010). Making sense of the meaning literature: an integrative review of meaning making and its effects on adjustment to stressful life events. Psychol. Bull. 136, 257–301. doi: 10.1037/a0018301, 20192563

[ref79] Park C. L. Ai A. L. (2006). Meaning making and growth: new directions for research on survivors of trauma. J. Loss Trauma 11, 389–407. doi: 10.1080/15325020600685295

[ref80] Park C. L. Mills M. A. Edmondson D. (2012). PTSD as meaning violation: testing a cognitive worldview perspective. Psychol. Trauma Theory Res. Pract. Policy 4, 66–73. doi: 10.1037/a0018792, 24860641 PMC4029350

[ref81] Parrish-Martin R. (2023) The creative arts personal growth group with centering prayer for sexual trauma survivors: a transformative-coping focused interpretative phenomenological analysis study. Doctoral dissertations and projects. Available online at: https://digitalcommons.liberty.edu/doctoral/4853

[ref82] Phanichrat T. Townshend J. M. (2010). Coping strategies used by survivors of childhood sexual abuse on the journey to recovery. J. Child Sex. Abuse 19, 62–78. doi: 10.1080/10538710903485617, 20390779

[ref83] Pooler D. K. Barros-Lane L. (2022). A national study of adult women sexually abused by clergy: insights for social workers. Soc. Work 67, 123–133. doi: 10.1093/sw/swac001, 35079841

[ref84] Preston A. M. Saigal S. Barrie R. McKinney H. Mooney S. Padala P. R. (2023). Defeated no more: meaning-making after military sexual trauma. Mil. Med. 188, e1483–e1489. doi: 10.1093/milmed/usab528, 35043959

[ref85] Raja S. Hasnain M. Hoersch M. Gove-Yin S. Rajagopalan C. (2015). Trauma informed care in medicine: current knowledge and future research directions. Fam. Community Health 38:216. doi: 10.1097/FCH.0000000000000071 26017000

[ref86] Ramakrishnan N. (2024). A qualitative investigation of unwanted arousal during rape. [dissertation] (Stillwater, Oklahoma: Oklahoma State University).

[ref87] Ratcliffe M. (2008). Feelings of Being: Phenomenology, Psychiatry and the sense of Reality. Oxford: Oxford University Press, ix–309.

[ref88] Saxena S. Oosterhoff P. (2023). Putting it behind? Young Indian victim-survivors of sexual violence and the quest for sexual well-being. Cult. Health Sex. 25, 1597–1611. doi: 10.1080/13691058.2023.2183989, 36876881

[ref89] Schuhmann C. Jacobs G. Damen A. (2026). “Meaning making as a process of orientation in life,” in Chaplaincy for a Plural World, eds. C. Schuhmann et al. (New York: Routledge), 3–22.

[ref90] Sicilia L. Capella C. Barrios M. Pereda N. (2024). Exploring the meanings of posttraumatic growth in Spanish survivors of clergy-perpetrated child sexual abuse: a phenomenological approach. J. Child Sex. Abus. 33, 3–25. doi: 10.1080/10538712.2024.2304241, 38229267

[ref91] Simon V. A. Feiring C. Kobielski McElroy S. (2010). Making meaning of traumatic events: youths’ strategies for processing childhood sexual abuse are associated with psychosocial adjustment. Child Maltreat. 15, 229–241. doi: 10.1177/1077559510370365, 20498128 PMC5496441

[ref92] Singh A. A. Garnett A. Williams D. (2013). Resilience strategies of African American women survivors of child sexual abuse: a qualitative inquiry. Counsel. Psychol. 41, 1093–1124. doi: 10.1177/0011000012469413

[ref93] Sinko L. (2019) Finding the strength to heal after campus unwanted sexual experiences: a journey of identity and strength [thesis]. Available online at: http://deepblue.lib.umich.edu/handle/2027.42/150062

[ref94] Smid G. E. Comtesse H. Boelen P. A. (2026). “Introduction,” in Psychotherapy for Prolonged and Traumatic Grief: A Guide for Mental Health Professionals, eds. Smid G. E. Comtesse H. Boelen P. A. (New York: Routledge/ Taylor & Francis), 1–10.

[ref95] Smith M. E. Kelly L. M. (2001). The journey of recovery after a rape experience. Issues Ment. Health Nurs. 22, 337–352. doi: 10.1080/01612840118791, 11885153

[ref96] Smith B. McGannon K. R. (2018). Developing rigor in qualitative research: problems and opportunities within sport and exercise psychology. Int. Rev. Sport Exerc. Psychol. 11, 101–121. doi: 10.1080/1750984X.2017.1317357

[ref97] Stavrova O. Luhmann M. (2016). Social connectedness as a source and consequence of meaning in life. J. Posit. Psychol. 11, 470–479. doi: 10.1080/17439760.2015.1117127

[ref98] Stephan A. (2012). Emotions, existential feelings, and their regulation. Emotion Rev. 4, 157–162. doi: 10.1177/1754073911430138

[ref99] Stidham A. W. Draucker C. B. Martsolf D. S. Mullen L. P. (2012). Altruism in survivors of sexual violence: the typology of helping others. J. Am. Psychiatr. Nurses Assoc. 18, 146–155. doi: 10.1177/1078390312440595, 22495915 PMC3947809

[ref100] Stoner-Harris T. (2024). Healing: reflections on my identity as a black female survivor of childhood sexual abuse. J. Feminist Fam. Ther. 36, 153–161. doi: 10.1080/08952833.2024.2389586

[ref101] Strauss Swanson C. Szymanski D. M. (2020). From pain to power: an exploration of activism, the #Metoo movement, and healing from sexual assault trauma. J. Couns. Psychol. 67, 653–668. doi: 10.1037/cou0000429, 32212761

[ref102] Tedeschi R. G. Calhoun L. G. (2004). Posttraumatic growth: conceptual foundations and empirical evidence. Psychol. Inq. 15, 1–18. doi: 10.1207/s15327965pli1501_01

[ref103] Thomas S. P. Hall J. M. (2008). Life trajectories of female child abuse survivors thriving in adulthood. Qual. Health Res. 18, 149–166. doi: 10.1177/1049732307312201, 18216336

[ref104] Tracy S. J. (2010). Qualitative quality: eight “big-tent” criteria for excellent qualitative research. Qual. Inq. 16, 837–851. doi: 10.1177/1077800410383121

[ref105] Tricco A. C. Lillie E. Zarin W. O’Brien K. K. Colquhoun H. Levac D. . (2018). PRISMA extension for scoping reviews (PRISMA-ScR): checklist and explanation. Ann. Intern. Med. 169, 467–473. doi: 10.7326/M18-0850 30178033

[ref106] Tummala-Narra P. Gonzalez L. D. Nguyen M. N. (2023). Experience of sexual violence among women of Mexican heritage raised in the United States. J. Cross-Cult. Psychol. 54, 385–406. doi: 10.1177/00220221221142867

[ref27] van der Werf J. (2022). Zichtbaar worden vanuit de schaduw: hoe het proces van zingeving een rol speelt in herstel na seksueel trauma [becoming visible from the shadows: how the process of meaning-making plays a role in recovery after sexual trauma]. Universiteit voor Humanistiek Research Portal. Available online at: https://research.uvh.nl/nl/studentTheses/zichtbaar-worden-vanuit-de-schaduw/ [Accessed April 18, 2023].

[ref107] van der Westhuizen M. Walker-Williams H. J. Fouché A. (2023). Meaning making mechanisms in women survivors of childhood sexual abuse: a scoping review. Trauma Violence Abuse 24, 1363–1386. doi: 10.1177/15248380211066100, 35109730

[ref108] van Wormer K. Berns L. (2004). The impact of priest sexual abuse: female survivors’ narratives. Affilia 19, 53–67. doi: 10.1177/0886109903260667

[ref109] Vazquez B. (2019). The role of religiosity, spirituality, and creative arts in the recovery process of Latina survivors of child sexual abuse. Theses and Dissertations. Available online at: https://digitalcommons.pepperdine.edu/etd/1088 [Accessed November 7, 2024].

[ref110] Vilenica S. Shakespeare-Finch J. Obst P. (2013). Exploring the process of meaning making in healing and growth after childhood sexual assault: a case study approach. Couns. Psychol. Q. 26, 39–54. doi: 10.1080/09515070.2012.728074

[ref111] Walker-Williams H. J. Fouché A. (2018). Resilience enabling processes and posttraumatic growth outcomes in a group of women survivors of childhood sexual abuse. Health SA Gesondheid 23:9. doi: 10.4102/hsag.v23i0.1134, 31934390 PMC6917442

[ref112] Wieberneit M. Thal S. Clare J. Notebaert L. Tubex H. (2024). Silenced survivors: a systematic review of the barriers to reporting, investigating, prosecuting, and sentencing of adult female rape and sexual assault. Trauma Violence Abuse 25, 3742–3757. doi: 10.1177/15248380241261404, 39077946 PMC11545439

[ref113] Wilson M. S. (2020) Resurrection through the voices of women who are survivors of childhood sexual abuse [Texas Christian University, Brite Divinity School]. Available online at: https://repository.tcu.edu/entities/publication/a72201a1-a2cf-4d0c-91e4-b3d9558af913 [Accessed November 7, 2024].

[ref114] Winterstein T. B. Avieli H. Gichaz M. (2023). Recovering the lost soul: older women’s reflections on past intrafamilial child sexual abuse. Qual. Health Res. 33, 426–439. doi: 10.1177/10497323231159802, 36882288

[ref115] Wright M. O. Crawford E. Sebastian K. (2007). Positive resolution of childhood sexual abuse experiences: the role of coping, benefit-finding and meaning-making. J. Fam. Violence 22, 597–608. doi: 10.1007/s10896-007-9111-1

[ref116] Wright C. Gabriel L. (2018). Perspectives of adult survivors of child sexual abuse: an exploration of the adjustments to self-structure through meaning-making in therapy. J. Child Sex. Abus. 27, 663–681. doi: 10.1080/10538712.2018.1496961, 30071187

[ref117] Yih C. (2024). Living in the aftermath: spiritual struggles of Hong Kong Christian women survivors of sexual violence. Pastoral Psychol. 73, 647–662. doi: 10.1007/s11089-024-01156-5

