# Supplementary material for: Meaning-making following sexual abuse: a scoping review and meta-synthesis
Source: Front Psychol. 2026 Mar 24;17:1798844. doi: 10.3389/fpsyg.2026.1798844 (PMC13055516; doi:10.3389/fpsyg.2026.1798844)
Supplement: Supplementary file 1 [file Table_1.DOCX]

*Supplementary Table 1*

Search strategies

*PsycInfo (Ovid) – 1/7/2025: APA PsycInfo <1806 to June 2025 Week 4>*

| # | Searches | Results |
| --- | --- | --- |
| 1 | exp Human Females/ or (Daughter* or Female* or Girl* or Mother* or Sister* or Widow or widows or wife or Wives or woman or women).mp. | 1503119 |
| 2 | exp Sex Offenses/ or Sexual Harassment/ or (clitorectomy or frottage or clitoridectomy or clitorolabiectomy or cybergrooming or frotteuris* or Incest* or infibulation or Rape or raped or rapes or reinfibulation or toucheuris* or (sex* adj2 (trauma* or abuse* or assault* or harass* or Offen* or traffick* or aggress* or bully* or coerc* or enslave* or exploit* or servitude or slave* or solicitat* or violen* or molestat* or victim*)) or (child* adj2 (prostitut* or molestat*)) or (clitoro adj2 labiectomy) or (genital* adj2 (mutilat* or circumci* or cut* or ritual*)) or ((female or pharaonic or woman or women) adj1 circumci*) or ((woman or women) adj infibulat*) or (clitor* adj3 (nicking or pricking)) or (bride* adj2 kidnap*) or ((force* or involuntary or coerc*) adj2 (marriage* or prostitut* or abortion* or pregnan* or reproduct*)) or (sabotage* adj2 (birth-control or contracepti*)) or (groom* adj3 (cyber or online or internet))).mp. | 81755 |
| 3 | exp Meaningfulness/ or (meaningful* or ((making or make or made or remaking or remake or find* or "in life" or attribut* or will) adj2 meaning*) or ((meaning* or attribut* or apprais* or accept*) adj3 (event* or incident* or trauma* or past)) or ((significan* or purpose*) adj3 (life or lives or task or tasks)) or ((global* or situation* or context* or subjective) adj2 (meaning* or sense* or apprais* or attribut*))).mp. | 121366 |
| 4 | exp goals/ or Personal Values/ or (goal* or motivation* or aspiration* or ((personal* or subject* or individual*) adj3 (value* or ethic* or moral* or attitude* or conviction*))).mp. | 496916 |
| 5 | exp World View/ or exp Ideology/ or Feminism/ or religion/ or exp religious beliefs/ or exp Spirituality/ or (Belief* or existential* or feminis* or ideolog* or philosoph* or political* or spiritual* or Faith* or religio* or (life adj3 (philosoph* or histor* or narrative*)) or (world* adj3 view*) or (God adj3 Concept*)).mp. | 538919 |
| 6 | exp "Resilience (Psychological)"/ or (resilien* or ((Emotional or Psychological or mental* or behavio*) adj3 (adapt* or Adjust* or stable or Stabilit* or flexib*)) or (positive adj3 (health* or psycholog*)) or (protect* adj3 factor*)).mp. | 208174 |
| 7 | exp Coping Behavior/ or (cope or coping or (runaway* adj2 (child* or adolescent* or behavio* or youth* or wom*)) or (self* adj2 handicap*) or (stress* adj2 manage*) or (growth adj3 (trauma* or posttraumatic or psycho*)) or (Lazarus adj1 theory) or (benefit* adj3 find*) or (emotion* adj3 eat*)).mp. | 156783 |
| 8 | "Recovery (Disorders)"/ or (Recover* or (Turning adj2 point*) or heal or heals or healed or healing* or healer* or restoration or resolution).mp. | 192902 |
| 9 | exp Systematic Review/ or exp Meta Analysis/ or exp "Treatment Guidelines"/ or (literature review or systematic review or meta analysis).md. or (((narrative or systematic or scoping or literature or integrative) adj2 review) or metaanalys* or meta-analys*).ab,ti. or (guideline* or recommendation* or cpg or framework* or protocol*).ti,ab. | 753501 |
| 10 | exp Treatment Effectiveness Evaluation/ or Clinical Trials/ or Mental Health Program Evaluation/ or Placebo/ or (placebo* or randomly or trial or (control* adj3 (trial* or study or studies or group*)) or factorial* or allocat* or assign* or volunteer* or crossover* or "cross over*").ti,ab. or (randomi* or ((singl* or doubl* or trebl* or tripl*) adj3 (blind* or mask* or dummy)) or (quasi adj5 (experimental or random*))).tw. | 587941 |
| 11 | (Clinical Trials/ or (((control or controlled) adj6 trial) or ((control or controlled) adj6 study) or ((control or controlled) adj1 active) or "open label*" or ((double or two or three or multi or trial) adj (arm or arms)) or (allocat* adj10 (arm or arms)) or placebo* or "sham-control*" or ((single or double or triple or assessor) adj1 (blind* or masked)) or nonrandom* or "non-random*" or "quasi-experimental" or crossover or "cross over" or "parallel group*" or "factorial trial").ti,ab,id. or (phase adj5 (study or trial)).ti,ab,id. or ((case* adj6 (matched or control*)) or (match* adj6 (pair or pairs or cohort* or control* or group* or healthy or age or sex or gender or patient* or subject* or participant*)) or (propensity adj6 (scor* or match*))).ti,ab,id. or (((Cohort Analysis/ or Followup Studies/ or Longitudinal Studies/ or Prospective Studies/ or Retrospective Studies/ or (cohort* or 'follow up' or followup or longitudina* or prospecti* or retrospecti* or observationa* or "cross sectiona*" or cross?ectional* or multicent* or 'multi-cent*' or consecutive*).ti,ab,id.) and ((group or groups or subgroup* or versus or vs or compar*).ti,ab,id. or ('odds ratio*' or 'relative odds' or 'risk ratio*' or 'relative risk*' or aor or arr or rrr).ab. or (("OR" or "RR") adj6 CI).ab.)) or (versus or vs or compar*).ti. or (compar* adj study).ti,ab,id.)) not ((comment* or editorial or letter).dt. or ((exp animals/ or exp Animal Models/ or animal.po.) not human.po.)) | 642209 |
| 12 | Case Report/ or ("Clinical Case Study" or "Nonclinical Case Study").md. or (case* or vignette* or casuistry or (clinical adj2 example*)).mp. | 679992 |
| 13 | exp Qualitative Methods/ or "Interview".dt. or exp Interviews/ or Narratives/ or Audiotapes/ or Digital Video/ or Videotapes/ or (((thematic or content) adj1 analys*) or focus-group* or ethnograph* or ethnograf* or etnograf* or field-stud* or phenomenolog* or narration* or narrative or (qualitative adj1 (stud* or analys* or research* or method*)) or multimethodolog* or mixed-method* or observation* or grounded-theory or ((audio or tape) adj1 recording*) or audiotape* or Videotape or ((semi-structured or semistructured or unstructured or informal or in-depth or indepth or face-to-face or structured or guide*) and (interview* or discussion* or questionnaire*))).mp. | 805281 |
| 14 | ("Brain Imaging" or "Clinical Case Study" or "Clinical Trial" or "Empirical Study" or "Experimental Replication" or "Field Study" or "Focus Group" or "Followup Study" or "Interview" or "Longitudinal Study" or "Mathematical Model" or "Nonclinical Case Study" or "Prospective Study" or "Qualitative Study" or "Quantitative Study" or "Retrospective Study" or "Scientific Simulation" or "Treatment Outcome" or "Twin Study").md. or (interview* or "Proof of Concept Study" or "brain imaging" or "Experimental Replication" or "focus group*" or "Mathematical Model" or "Scientific Simulation" or "Treatment Outcome" or ((Empirical or Behavior* or Behaviour* or Applied or Population* or Descriptive or Clinical or Field or Followup or Follow-up or Longitudinal or Nonclinical or prospective* or Qualitative or Quantitative or Investigat* or Retrospective or Case or Observation* or twin) adj4 (Study or Studies or Research or Report or Reports or trial*)) or (Grounded adj4 Theor*)).ti,ab,id. | 3644682 |
| 15 | 1 and 2 and (3 or 4 or 5 or 6 or 7) and 8 and (9 or 10 or 11 or 12 or 13 or 14) | 1023 |

*Ovid Medline – 01/07/2025: Ovid MEDLINE(R) ALL <1946 to June 30, 2025>*

| # | Searches | Results |
| --- | --- | --- |
| 1 | exp Women/ or (Daughter* or Female* or Girl* or Mother* or Sister* or Widow or widows or wife or Wives or woman or women).mp. | 10916093 |
| 2 | exp Sex Offenses/ or Sexual Harassment/ or (clitorectomy or frottage or clitoridectomy or clitorolabiectomy or cybergrooming or frotteuris* or Incest* or infibulation or Rape or raped or rapes or reinfibulation or toucheuris* or (sex* adj2 (trauma* or abuse* or assault* or harass* or Offen* or traffick* or aggress* or bully* or coerc* or enslave* or exploit* or servitude or slave* or solicitat* or violen* or molestat* or victim*)) or (child* adj2 (prostitut* or molestat*)) or (clitoro adj2 labiectomy) or (genital* adj2 (mutilat* or circumci* or cut* or ritual*)) or ((female or pharaonic or woman or women) adj1 circumci*) or ((woman or women) adj infibulat*) or (clitor* adj3 (nicking or pricking)) or (bride* adj2 kidnap*) or ((force* or involuntary or coerc*) adj2 (marriage* or prostitut* or abortion* or pregnan* or reproduct*)) or (sabotage* adj2 (birth-control or contracepti*)) or (groom* adj3 (cyber or online or internet))).mp. | 62674 |
| 3 | (meaningful* or ((making or make or made or remaking or remake or find* or "in life" or attribut* or will) adj2 meaning*) or ((meaning* or attribut* or apprais* or accept*) adj3 (event* or incident* or trauma* or past)) or ((significan* or purpose*) adj3 (life or lives or task or tasks)) or ((global* or situation* or context* or subjective) adj2 (meaning* or sense* or apprais* or attribut*))).mp. | 168330 |
| 4 | goals/ or (goal* or motivation* or aspiration* or ((personal* or subject* or individual*) adj3 (value* or ethic* or moral* or attitude* or conviction*))).mp. | 812682 |
| 5 | Feminism/ or religion/ or Spirituality/ or (Belief* or existential* or feminis* or ideolog* or philosoph* or political* or spiritual* or Faith* or religio* or (life adj3 (philosoph* or histor* or narrative*)) or (world* adj3 view*) or (God adj3 Concept*)).mp. | 390996 |
| 6 | exp "Resilience, Psychological"/ or (resilien* or ((Emotional or Psychological or mental* or behavio*) adj3 (adapt* or Adjust* or stable or Stabilit* or flexib*)) or (positive adj3 (health* or psycholog*)) or (protect* adj3 factor*)).mp. | 298077 |
| 7 | "Adaptation, Psychological"/ or exp Emotional Adjustment/ or (cope or coping or (runaway* adj2 (child* or adolescent* or behavio* or youth* or wom*)) or (self* adj2 handicap*) or (stress* adj2 manage*) or (growth adj3 (trauma* or posttraumatic or psycho*)) or (Lazarus adj1 theory) or (benefit* adj3 find*) or (emotion* adj3 eat*)).mp. | 218225 |
| 8 | Mental Health Recovery/ or (Recover* or (Turning adj2 point*) or heal or heals or healed or healing* or healer* or restoration or resolution).mp. | 1946856 |
| 9 | Systematic Review/ or "meta analysis (topic)"/ or exp Meta-Analysis/ or exp practice guideline/ or (Systematic Review or Meta-Analysis).pt. or (((narrative or systematic or scoping or literature or integrative) adj2 review) or metaanalys* or meta-analys*).ab,ti. or (guideline* or recommendation* or cpg or framework* or protocol*).ti,ab. | 2618321 |
| 10 | exp Clinical Trial/ or exp "Evaluation Studies as Topic"/ or exp Evaluation Studies/ or exp Placebo Effect/ or "Evaluation Stud*".pt. or "randomized controlled trial*".pt. or "clinical trial*".pt. or (placebo* or randomly or trial or (control* adj3 (trial* or study or studies or group*)) or factorial* or allocat* or assign* or volunteer* or crossover* or "cross over*").ti,ab. or (randomi* or ((singl* or doubl* or trebl* or tripl*) adj3 (blind* or mask* or dummy)) or (quasi adj5 (experimental or random*))).tw. | 4664580 |
| 11 | (controlled clinical trial/ or (((control or controlled) adj6 trial) or ((control or controlled) adj6 study) or ((control or controlled) adj1 active) or "open label*" or ((double or two or three or multi or trial) adj (arm or arms)) or (allocat* adj10 (arm or arms)) or placebo* or "sham-control*" or ((single or double or triple or assessor) adj1 (blind* or masked)) or nonrandom* or "non-random*" or "quasi-experimental" or crossover or "cross over" or "parallel group*" or "factorial trial").ti,ab,kf. or clinical trial, phase ii/ or clinical trial, phase iii/ or clinical trial, phase iv/ or (phase adj5 (study or trial)).ti,ab,kf. or (Case-Control Studies/ or ((case* adj6 (matched or control*)) or (match* adj6 (pair or pairs or cohort* or control* or group* or healthy or age or sex or gender or patient* or subject* or participant*))).ti,ab,kf. or (propensity adj6 (scor* or match*)).ti,ab,kf.) or (((exp cohort studies/ or observational study/ or cross-sectional studies/ or multicenter study/ or (cohort* or 'follow up' or followup or longitudinal* or prospective* or retrospective* or observational* or cross sectional* or cross?ectional* or multicent* or 'multi-cent*' or consecutive*).ti,ab,kf.) and ((group or groups or subgroup* or versus or vs or compar*).ti,ab,kf. or ('odds ratio*' or 'relative odds' or 'risk ratio*' or 'relative risk*' or aor or arr or rrr).ab. or (("OR" or "RR") adj6 CI).ab.)) or (versus or vs or compar*).ti. or Comparative study/ or (compar* adj study).ti,ab,kf. or historically controlled study/)) not ((exp animals/ or exp models,animal/) not humans/) not (letter/ or comment/ or editorial/) | 5485042 |
| 12 | Case Reports/ or Case Reports.pt. or Single-Case Studies as Topic/ or (case* or vignette* or casuistry or (clinical adj2 example*)).mp. | 5920757 |
| 13 | exp Qualitative Research/ or Interview.pt. or Interviews as Topic/ or Narration/ or Personal Narratives as Topic/ or focus groups/ or interviews as topic/ or (((thematic or content) adj1 analys*) or focus-group* or ethnograph* or ethnograf* or etnograf* or field-stud* or phenomenolog* or narration* or narrative or (qualitative adj1 (stud* or analys* or research* or method*)) or multimethodolog* or mixed-method* or observation* or grounded-theory or ((audio or tape) adj1 recording*) or audiotape* or Videotape or ((semi-structured or semistructured or unstructured or informal or in-depth or indepth or face-to-face or structured or guide*) and (interview* or discussion* or questionnaire*))).mp. | 1999532 |
| 14 | ("Brain Imaging" or "Clinical Case Study" or "Clinical Trial" or "Empirical Study" or "Experimental Replication" or "Field Study" or "Focus Group" or "Followup Study" or "Interview" or "Longitudinal Study" or "Mathematical Model" or "Nonclinical Case Study" or "Prospective Study" or "Qualitative Study" or "Quantitative Study" or "Retrospective Study" or "Scientific Simulation" or "Treatment Outcome" or "Twin Study").pt. or (interview* or "Proof of Concept Study" or "brain imaging" or "Experimental Replication" or "focus group*" or "Mathematical Model" or "Scientific Simulation" or "Treatment Outcome" or ((Empirical or Behavior* or Behaviour* or Applied or Population* or Descriptive or Clinical or Field or Followup or Follow-up or Longitudinal or Nonclinical or prospective* or Qualitative or Quantitative or Investigat* or Retrospective or Case or Observation* or twin) adj4 (Study or Studies or Research or Report or Reports or trial*)) or (Grounded adj4 Theor*)).ti,ab,kf,pt. | 6190380 |
| 15 | 1 and 2 and (3 or 4 or 5 or 6 or 7) and 8 and (9 or 10 or 11 or 12 or 13 or 14) | 369 |

*Embase (Ovid) – 1/7/2025: Embase <1974 to 2025 Week 26>*

| # | Searches | Results |
| --- | --- | --- |
| 1 | female/ or exp female by marital status/ or exp female by occupation/ or exp female by sexual orientation/ or girl/ or exp named groups by pregnancy/ or (Daughter* or Female* or Girl* or Mother* or Sister* or Widow or widows or wife or Wives or woman or women).mp. | 13895143 |
| 2 | sexual crime/ or exp sexual violence/ or (clitorectomy or frottage or clitoridectomy or clitorolabiectomy or cybergrooming or frotteuris* or Incest* or infibulation or Rape or raped or rapes or reinfibulation or toucheuris* or (sex* adj2 (trauma* or abuse* or assault* or harass* or Offen* or traffick* or aggress* or bully* or coerc* or enslave* or exploit* or servitude or slave* or solicitat* or violen* or molestat* or victim*)) or (child* adj2 (prostitut* or molestat*)) or (clitoro adj2 labiectomy) or (genital* adj2 (mutilat* or circumci* or cut* or ritual*)) or ((female or pharaonic or woman or women) adj1 circumci*) or ((woman or women) adj infibulat*) or (clitor* adj3 (nicking or pricking)) or (bride* adj2 kidnap*) or ((force* or involuntary or coerc*) adj2 (marriage* or prostitut* or abortion* or pregnan* or reproduct*)) or (sabotage* adj2 (birth-control or contracepti*)) or (groom* adj3 (cyber or online or internet))).mp. | 86889 |
| 3 | exp meaning-making/ or (meaningful* or ((making or make or made or remaking or remake or find* or "in life" or attribut* or will) adj2 meaning*) or ((meaning* or attribut* or apprais* or accept*) adj3 (event* or incident* or trauma* or past)) or ((significan* or purpose*) adj3 (life or lives or task or tasks)) or ((global* or situation* or context* or subjective) adj2 (meaning* or sense* or apprais* or attribut*))).mp. | 234324 |
| 4 | exp motivation/ or Personal Value/ or (goal* or motivation* or aspiration* or ((personal* or subject* or individual*) adj3 (value* or ethic* or moral* or attitude* or conviction*))).mp. | 1381543 |
| 5 | exp Ideology/ or Feminism/ or religion/ or (Belief* or existential* or feminis* or ideolog* or philosoph* or political* or spiritual* or Faith* or religio* or (life adj3 (philosoph* or histor* or narrative*)) or (world* adj3 view*) or (God adj3 Concept*)).mp. | 484379 |
| 6 | exp psychological resilience/ or (resilien* or ((Emotional or Psychological or mental* or behavio*) adj3 (adapt* or Adjust* or stable or Stabilit* or flexib*)) or (positive adj3 (health* or psycholog*)) or (protect* adj3 factor*)).mp. | 293853 |
| 7 | exp coping behavior/ or (cope or coping or (runaway* adj2 (child* or adolescent* or behavio* or youth* or wom*)) or (self* adj2 handicap*) or (stress* adj2 manage*) or (growth adj3 (trauma* or posttraumatic or psycho*)) or (Lazarus adj1 theory) or (benefit* adj3 find*) or (emotion* adj3 eat*)).mp. | 220897 |
| 8 | healing/ or (Recover* or (Turning adj2 point*) or heal or heals or healed or healing* or healer* or restoration or resolution).mp. | 2499695 |
| 9 | Systematic Review/ or "meta analysis (topic)"/ or exp Meta-Analysis/ or exp practice guideline/ or (((narrative or systematic or scoping or literature or integrative) adj2 review) or metaanalys* or meta-analys*).ab,ti. or (guideline* or recommendation* or cpg or framework* or protocol*).ti,ab. | 3962397 |
| 10 | exp clinical trial/ or exp "Evaluation Studies as Topic"/ or exp Evaluation Studies/ or exp Placebo Effect/ or (placebo* or randomly or trial or (control* adj3 (trial* or study or studies or group*)) or factorial* or allocat* or assign* or volunteer* or crossover* or "cross over*").ti,ab. or (randomi* or ((singl* or doubl* or trebl* or tripl*) adj3 (blind* or mask* or dummy)) or (quasi adj5 (experimental or random*))).tw. | 5857742 |
| 11 | (exp "clinical trial (topic)"/ or exp clinical trial/ or (((control or controlled) adj6 trial) or ((control or controlled) adj6 study) or ((control or controlled) adj1 active) or "open label*" or ((double or two or three or multi or trial) adj (arm or arms)) or (allocat* adj10 (arm or arms)) or placebo* or "sham-control*" or ((single or double or triple or assessor) adj1 (blind* or masked)) or nonrandom* or "non-random*" or "quasi-experimental" or crossover or "cross over" or "parallel group*" or "factorial trial").ti,ab,kf. or (phase adj5 (study or trial)).ti,ab,kf. or ((case* adj6 (matched or control*)) or (match* adj6 (pair or pairs or cohort* or control* or group* or healthy or age or sex or gender or patient* or subject* or participant*)) or (propensity adj6 (scor* or match*))).ti,ab,kf. or (((Cohort Analysis/ or Follow up/ or exp longitudinal study/ or Prospective Study/ or Retrospective Study/ or multicenter study/ or cross-sectional study/ or (cohort* or 'follow up' or followup or longitudina* or prospecti* or retrospecti* or observationa* or "cross sectiona*" or cross?ectional* or multicent* or 'multi-cent*' or consecutive*).ti,ab,kf.) and ((group or groups or subgroup* or versus or vs or compar*).ti,ab,kf. or ('odds ratio*' or 'relative odds' or 'risk ratio*' or 'relative risk*' or aor or arr or rrr).ab. or (("OR" or "RR") adj6 CI).ab.)) or (versus or vs or compar*).ti. or (compar* adj study).ti,ab,kf.)) not ((comment* or editorial or letter).pt. or ((exp animal/ or exp Animal Model/) not human/)) | 8180124 |
| 12 | case report/ or exp case study/ or (case* or vignette* or casuistry or (clinical adj2 example*)).mp. | 7542094 |
| 13 | qualitative analysis/ or exp qualitative research/ or exp Interview/ or Narrative/ or exp recording/ or (((thematic or content) adj1 analys*) or focus-group* or ethnograph* or ethnograf* or etnograf* or field-stud* or phenomenolog* or narration* or narrative or (qualitative adj1 (stud* or analys* or research* or method*)) or multimethodolog* or mixed-method* or observation* or grounded-theory or ((audio or tape) adj1 recording*) or audiotape* or Videotape or ((semi-structured or semistructured or unstructured or informal or in-depth or indepth or face-to-face or structured or guide*) and (interview* or discussion* or questionnaire*))).mp. | 3018614 |
| 14 | (interview* or "Proof of Concept Study" or "brain imaging" or "Experimental Replication" or "focus group*" or "Mathematical Model" or "Scientific Simulation" or "Treatment Outcome" or ((Empirical or Behavior* or Behaviour* or Applied or Population* or Descriptive or Clinical or Field or Followup or Follow-up or Longitudinal or Nonclinical or prospective* or Qualitative or Quantitative or Investigat* or Retrospective or Case or Observation* or twin) adj4 (Study or Studies or Research or Report or Reports or trial*)) or (Grounded adj4 Theor*)).ti,ab,kf. | 7954139 |
| 15 | 1 and 2 and (3 or 4 or 5 or 6 or 7) and 8 and (9 or 10 or 11 or 12 or 13 or 14) | 550 |

*Ovid Evidence Based Medicine Reviews – 2/7/2025: All EBM Reviews - Cochrane DSR, ACP Journal Club, DARE, CCA, CCTR, CMR, HTA, and NHSEED*

| # | Searches | Results |
| --- | --- | --- |
| 1 | (Daughter* or Female* or Girl* or Mother* or Sister* or Widow or widows or wife or Wives or woman or women).mp. | 1107920 |
| 2 | (clitorectomy or frottage or clitoridectomy or clitorolabiectomy or cybergrooming or frotteuris* or Incest* or infibulation or Rape or raped or rapes or reinfibulation or toucheuris* or (sex* adj2 (trauma* or abuse* or assault* or harass* or Offen* or traffick* or aggress* or bully* or coerc* or enslave* or exploit* or servitude or slave* or solicitat* or violen* or molestat* or victim*)) or (child* adj2 (prostitut* or molestat*)) or (clitoro adj2 labiectomy) or (genital* adj2 (mutilat* or circumci* or cut* or ritual*)) or ((female or pharaonic or woman or women) adj1 circumci*) or ((woman or women) adj infibulat*) or (clitor* adj3 (nicking or pricking)) or (bride* adj2 kidnap*) or ((force* or involuntary or coerc*) adj2 (marriage* or prostitut* or abortion* or pregnan* or reproduct*)) or (sabotage* adj2 (birth-control or contracepti*)) or (groom* adj3 (cyber or online or internet))).mp. | 3254 |
| 3 | (meaningful* or ((making or "make" or made or remaking or remake or find* or "in life" or attribut* or will) adj2 meaning*) or ((meaning* or attribut* or apprais* or accept*) adj3 (event* or incident* or trauma* or past)) or ((significan* or purpose*) adj3 (life or lives or task or tasks)) or ((global* or situation* or context* or subjective) adj2 (meaning* or sense* or apprais* or attribut*))).mp. | 34475 |
| 4 | (goal* or motivation* or aspiration* or ((personal* or subject* or individual*) adj3 (value* or ethic* or moral* or attitude* or conviction*))).mp. | 100799 |
| 5 | (Belief* or existential* or feminis* or ideolog* or philosoph* or political* or spiritual* or Faith* or religio* or (life adj3 (philosoph* or histor* or narrative*)) or (world* adj3 view*) or (God adj3 Concept*)).mp. | 22435 |
| 6 | (resilien* or ((Emotional or Psychological or mental* or behavio*) adj3 (adapt* or Adjust* or stable or Stabilit* or flexib*)) or (positive adj3 (health* or psycholog*)) or (protect* adj3 factor*)).mp. | 21637 |
| 7 | (cope or coping or (runaway* adj2 (child* or adolescent* or behavio* or youth* or wom*)) or (self* adj2 handicap*) or (stress* adj2 manage*) or (growth adj3 (trauma* or posttraumatic or psycho*)) or (Lazarus adj1 theory) or (benefit* adj3 find*) or (emotion* adj3 eat*)).mp. | 23093 |
| 8 | (Recover* or (Turning adj2 point*) or heal or heals or healed or healing* or healer* or restoration or resolution).mp. | 167832 |
| 9 | review.dt. or (((narrative or systematic or scoping or literature or integrative) adj2 review) or metaanalys* or meta-analys*).ab,ti,dt,pt. or (guideline* or recommendation* or cpg or framework* or protocol*).ti,ab,dt,pt. | 296989 |
| 10 | (Evaluation* or Trial*).dt,pt. or (placebo* or randomly or trial or (control* adj3 (trial* or study or studies or group*)) or factorial* or allocat* or assign* or volunteer* or crossover* or "cross over*").ti,ab. or (randomi* or ((singl* or doubl* or trebl* or tripl*) adj3 (blind* or mask* or dummy)) or (quasi adj5 (experimental or random*))).tw. | 1901565 |
| 11 | (trial*.dt,pt. or (((control or controlled) adj6 trial) or ((control or controlled) adj6 study) or ((control or controlled) adj1 active) or "open label*" or ((double or two or three or multi or trial) adj (arm or arms)) or (allocat* adj10 (arm or arms)) or placebo* or "sham-control*" or ((single or double or triple or assessor) adj1 (blind* or masked)) or nonrandom* or "non-random*" or "quasi-experimental" or crossover or "cross over" or "parallel group*" or "factorial trial").ti,ab,kw. or (phase adj5 (study or trial)).ti,ab,kw. or ((case* adj6 (matched or control*)) or (match* adj6 (pair or pairs or cohort* or control* or group* or healthy or age or sex or gender or patient* or subject* or participant*)) or (propensity adj6 (scor* or match*))).ti,ab,kw. or (((cohort* or 'follow up' or followup or longitudina* or prospecti* or retrospecti* or observationa* or "cross sectiona*" or cross?ectional* or multicent* or 'multi-cent*' or consecutive*).ti,ab,kw,dt,pt. and ((group or groups or subgroup* or versus or vs or compar*).ti,ab,kw. or ('odds ratio*' or 'relative odds' or 'risk ratio*' or 'relative risk*' or aor or arr or rrr).ab. or (("OR" or "RR") adj6 CI).ab.)) or (versus or vs or compar*).ti. or (compar* adj study).ti,ab,kw.)) not (comment* or editorial or letter).dt,pt. | 1851420 |
| 12 | (case* or vignette* or casuistry or (clinical adj2 example*)).mp,dt,pt. | 214045 |
| 13 | (((thematic or content) adj1 analys*) or focus-group* or ethnograph* or ethnograf* or etnograf* or field-stud* or phenomenolog* or narration* or narrative or (qualitative adj1 (stud* or analys* or research* or method*)) or multimethodolog* or mixed-method* or observation* or grounded-theory or ((audio or tape) adj1 recording*) or audiotape* or Videotape or ((semi-structured or semistructured or unstructured or informal or in-depth or indepth or face-to-face or structured or guide*) and (interview* or discussion* or questionnaire*))).mp,dt,pt. | 190853 |
| 14 | (interview* or "Proof of Concept Study" or "brain imaging" or "Experimental Replication" or "focus group*" or "Mathematical Model" or "Scientific Simulation" or "Treatment Outcome" or ((Empirical or Behavior* or Behaviour* or Applied or Population* or Descriptive or Clinical or Field or Followup or Follow-up or Longitudinal or Nonclinical or prospective* or Qualitative or Quantitative or Investigat* or Retrospective or Case or Observation* or twin) adj4 (Study or Studies or Research or Report or Reports or trial*)) or (Grounded adj4 Theor*)).ti,ab,kw,dt,pt. | 1045236 |
| 15 | 1 and 2 and (3 or 4 or 5 or 6 or 7) and 8 and (9 or 10 or 11 or 12 or 13 or 14) | 124 |

*PTSDpubs - 2/7/2025*

| Set# | Searched for | Results |
| --- | --- | --- |
| S1 | MAINSUBJECT.EXACT("Females") AND (MAINSUBJECT.EXACT("World Assumptions") OR MAINSUBJECT.EXACT("Spirituality") OR MAINSUBJECT.EXACT("Resilience") OR MAINSUBJECT.EXACT.EXPLODE("Coping Behavior") OR TI,AB,SU(meaningful* OR ((making OR make OR made OR remaking OR remake OR find* OR "in life" OR attribut* OR will) NEAR/2 meaning*) OR ((meaning* OR attribut* OR apprais* OR accept*) NEAR/3 (event* OR incident* OR trauma* OR past)) OR ((significan* OR purpose*) NEAR/3 (life OR lives OR task OR tasks)) OR ((global* OR situation* OR context* OR subjective) NEAR/2 (meaning* OR sense* OR apprais* OR attribut*)) OR goal* OR (personal* NEAR/3 value* ) OR Belief* OR feminis* OR ideolog* OR philosoph* OR Faith* OR religio*)) AND TI,AB,SU(Recover* OR (Turning NEAR/2 point*) OR heal OR heals Or healed OR healing* OR healer* OR restoration OR resolution) | 392 |

*Web of Science– 2/7/2025 – Entitlements: WOS.SCI: 1900 to 2025, WOS.AHCI: 1975 to 2025, WOS.ESCI: 2020 to 2025, WOS.SSCI: 1900 to 2025*

| # | Search Query | Results |
| --- | --- | --- |
| 1 | TS=(Daughter* OR Female* OR Girl* OR Mother* OR Sister* OR Widow OR widows OR wife OR Wives OR woman OR women ) | 3780955 |
| 2 | TS=(clitorectomy OR frottage OR clitoridectomy OR clitorolabiectomy OR cybergrooming OR frotteuris* OR Incest* OR infibulation OR Rape OR raped OR rapes OR reinfibulation OR toucheuris* OR (sex* NEAR/2 (trauma* OR abuse* OR assault* OR harass* OR Offen* OR traffick* OR aggress* OR bully* OR coerc* OR enslave* OR exploit* OR servitude OR slave* OR solicitat* OR violen* OR molestat* OR victim*)) OR (child* NEAR/2 (prostitut* OR molestat* )) OR (clitoro NEAR/2 labiectomy) OR (genital* NEAR/2 (mutilat* OR circumci* OR cut* OR ritual*)) OR ((female OR pharaonic OR woman OR women) NEAR/1 circumci*) OR ((woman OR women) NEAR/1 infibulat*) OR (clitor* NEAR/3 (nicking OR pricking)) OR (bride* NEAR/2 kidnap*) OR ((force* OR involuntary OR coerc*) NEAR/2 ( marriage* OR prostitut* OR abortion* OR pregnan* OR reproduct*)) OR (sabotage* NEAR/2 (birth-control OR contracepti*)) OR (groom* NEAR/3 (cyber OR online OR internet)) ) | 115052 |
| 3 | TS=(meaningful* OR ((making OR make OR made OR remaking OR remake OR find* OR "in life" OR attribut* OR will) NEAR/2 meaning*) OR ((meaning* OR attribut* OR apprais* OR accept*) NEAR/3 (event* OR incident* OR trauma* OR past)) OR ((significan* OR purpose*) NEAR/3 (life OR lives OR task OR tasks)) OR ((global* OR situation* OR context* OR subjective) NEAR/2 (meaning* OR sense* OR apprais* OR attribut*)) ) | 337714 |
| 4 | TS=(goal* OR motivation* OR aspiration* OR ((personal* or subject* OR individual*) NEAR/3 (value* OR ethic* OR moral* OR attitude* OR conviction*))) | 1405940 |
| 5 | TS=(Belief* OR existential* OR feminis* OR ideolog* OR philosoph* OR political* OR spiritual* OR Faith* OR religio* OR (life NEAR/3 (philosoph* OR histor* OR narrative*)) OR (world* NEAR/3 view*) OR (God NEAR/3 Concept*)) | 1601434 |
| 6 | TS=(resilien* or ((Emotional OR Psychological OR mental* OR behavio*) NEAR/3 (adapt* OR Adjust* OR stable OR Stabilit* OR flexib* )) or (positive NEAR/3 (health* OR psycholog*)) OR (protect* NEAR/3 factor*) ) | 449282 |
| 7 | TS=(cope OR coping OR (runaway* NEAR/2 (child* OR adolescent* OR behavio* OR youth* OR wom*)) OR (self* NEAR/2 handicap*) OR (stress* NEAR/2 manage*) OR (growth NEAR/3 (trauma* OR posttraumatic OR psycho*)) OR (Lazarus NEAR/1 theory) OR (benefit* NEAR/3 find*) OR (emotion* NEAR/3 eat*) ) | 266151 |
| 8 | TS=(Recover* OR (Turning NEAR/2 point*) OR heal OR heals Or healed OR healing* OR healer* OR restoration OR resolution) | 3420734 |
| 9 | TS= (((narrative OR systematic OR scoping OR literature OR integrative) NEAR/2 review) or metaanalys* or meta-analys* or guideline* or recommendation* or cpg or framework* or protocol*) | 4907358 |
| 10 | TS=(placebo* OR randomly OR trial OR (control* NEAR/3 (trial* OR study OR studies OR group*)) OR factorial* OR allocat* OR assign* OR volunteer* OR crossover* OR "cross over*" OR randomi* OR ((singl* OR doubl* OR trebl* OR tripl*) NEAR/3 (blind* OR mask* OR dummy)) OR (quasi NEAR/5 (experimental OR random*))) | 5038633 |
| 11 | TS= (random* or control* or study or trial or compar* or group or groups or therapy or treatment or intervention) | 38181612 |
| 12 | TS= (case* OR vignette* OR casuistry OR (clinical NEAR/2 example*) ) | 6255657 |
| 13 | TS= (((thematic OR content) NEAR/1 analys*) OR focus-group* OR ethnograph* OR ethnograf* OR etnograf* OR field-stud* OR phenomenolog* OR narration* OR narrative OR (qualitative NEAR/1 (stud* OR analys* OR research* OR method*)) OR multimethodolog* OR mixed-method* OR observation* OR grounded-theory OR ((audio OR tape) NEAR/1 recording*) OR audiotape* OR Videotape OR ((semi-structured OR semistructured OR unstructured OR informal OR in-depth OR indepth OR face-to-face OR structured OR guide*) AND (interview* OR discussion* OR questionnaire*)) ) | 3606345 |
| 14 | TS= (interview* OR "Proof of Concept Study" OR "brain imaging" or "Experimental Replication" OR "focus group*" OR "Mathematical Model" OR "Scientific Simulation" OR "Treatment Outcome" OR ((Empirical or Behavior* or Behaviour* or Applied or Population* or Descriptive or Clinical or Field or Followup OR Follow-up OR Longitudinal OR Nonclinical or prospective* OR Qualitative or Quantitative or Investigat* or Retrospective OR Case or Observation* OR twin) NEAR/4 (Study or Studies or Research or Report or Reports OR trial*)) or (Grounded NEAR/4 Theor*)) | 8621527 |
| 15 | #1 and #2 and (#3 or #4 or #5 or #6 or #7) and #8 and (#9 or #10 or #11 or #12 or #13 or #14) | 658 |

*Supplementary Table 2*

Overview of numbers of retrieved and duplicate references

| Database | Platform | Coverage | Number retrieved | Duplicates before export | New articles added to screening | Duplicates during screening | Total duplicates | New total screened |
| --- | --- | --- | --- | --- | --- | --- | --- | --- |
| PsycInfo | Ovid | 1806 – present | 1023 | 49 | 974 | 0 | 49 | 974 |
| Ovid Medline | Ovid | 1946 – present | 369 | 178 | 191 |  | 178 | 191 |
| Embase | Ovid | 1947 – present | 550 | 315 | 235 | 1 | 316 | 234 |
| Ovid Evidence Based Medicine Reviews | Ovid | Cochrane DSR 2005 - present, ACP Journal Club 1991 - present, DARE 1991-2015, CCA 2012 - present, CCTR 1991 - present, CMR 1995-2012, HTA 2001-2016, NHSEED 1995-2015 | 124 | 16 | 108 |  | 16 | 108 |
| PTSDpubs | ProQuest | 1871 – present | 392 | 77 | 315 | 1 | 78 | 314 |
| Web of Science | Clarivate | 1900 – present | 658 | 321 | 337 |  | 321 | 337 |
| Totals | | | 3116 | 956 | 2160 | 2 | 958 | 2158 |

*Supplementary Table 3.*

Preferred Reporting Items for Systematic reviews and Meta-Analyses extension for Scoping Reviews (PRISMA-ScR) Checklist

| SECTION | ITEM | PRISMA-ScR CHECKLIST ITEM | REPORTED ON PAGE # |
| --- | --- | --- | --- |
| **TITLE** | | | |
| Title | 1 | Identify the report as a scoping review. | 1 |
| **ABSTRACT** | | | |
| Structured summary | 2 | Provide a structured summary that includes (as applicable): background, objectives, eligibility criteria, sources of evidence, charting methods, results, and conclusions that relate to the review questions and objectives. | 2-3 |
| **INTRODUCTION** | | | |
| Rationale | 3 | Describe the rationale for the review in the context of what is already known. Explain why the review questions/objectives lend themselves to a scoping review approach. | 5, 6 |
| Objectives | 4 | Provide an explicit statement of the questions and objectives being addressed with reference to their key elements (e.g., population or participants, concepts, and context) or other relevant key elements used to conceptualize the review questions and/or objectives. | 5 |
| **METHODS** | | | |
| Protocol and registration | 5 | Indicate whether a review protocol exists; state if and where it can be accessed (e.g., a Web address); and if available, provide registration information, including the registration number. | 6 |
| Eligibility criteria | 6 | Specify characteristics of the sources of evidence used as eligibility criteria (e.g., years considered, language, and publication status), and provide a rationale. | 6 |
| Information sources* | 7 | Describe all information sources in the search (e.g., databases with dates of coverage and contact with authors to identify additional sources), as well as the date the most recent search was executed. | 6 and Supplementary Tables |
| Search | 8 | Present the full electronic search strategy for at least 1 database, including any limits used, such that it could be repeated. | Supplementary Tables |
| Selection of sources of evidence† | 9 | State the process for selecting sources of evidence (i.e., screening and eligibility) included in the scoping review. | 7-8 |
| Data charting process‡ | 10 | Describe the methods of charting data from the included sources of evidence (e.g., calibrated forms or forms that have been tested by the team before their use, and whether data charting was done independently or in duplicate) and any processes for obtaining and confirming data from investigators. | 7-8 |
| Data items | 11 | List and define all variables for which data were sought and any assumptions and simplifications made. | 8 |
| Critical appraisal of individual sources of evidence§ | 12 | If done, provide a rationale for conducting a critical appraisal of included sources of evidence; describe the methods used and how this information was used in any data synthesis (if appropriate). | 9 |
| Synthesis of results | 13 | Describe the methods of handling and summarizing the data that were charted. | 8-10 |
| **RESULTS** | | | |
| Selection of sources of evidence | 14 | Give numbers of sources of evidence screened, assessed for eligibility, and included in the review, with reasons for exclusions at each stage, ideally using a flow diagram. | 7, Figure 1, Supplementary Table 1 and 2 |
| Characteristics of sources of evidence | 15 | For each source of evidence, present characteristics for which data were charted and provide the citations. | Tables 1, 2, and 3 |
| Critical appraisal within sources of evidence | 16 | If done, present data on critical appraisal of included sources of evidence (see item 12). | N/A |
| Results of individual sources of evidence | 17 | For each included source of evidence, present the relevant data that were charted that relate to the review questions and objectives. | 8-30 |
| Synthesis of results | 18 | Summarize and/or present the charting results as they relate to the review questions and objectives. | Figure 2 |
| **DISCUSSION** | | | |
| Summary of evidence | 19 | Summarize the main results (including an overview of concepts, themes, and types of evidence available), link to the review questions and objectives, and consider the relevance to key groups. | 31-34, Figure 3 |
| Limitations | 20 | Discuss the limitations of the scoping review process. | 34 |
| Conclusions | 21 | Provide a general interpretation of the results with respect to the review questions and objectives, as well as potential implications and/or next steps. | 31, 34-36 |
| **FUNDING** | | | |
| Funding | 22 | Describe sources of funding for the included sources of evidence, as well as sources of funding for the scoping review. Describe the role of the funders of the scoping review. | 36 |

*Note.* Table source: Tricco et al. (2018).

Reference

Tricco, A. C., Lillie, E., Zarin, W., O’Brien, K. K., Colquhoun, H., Levac, D., Moher, D., Peters, M. D. J., Horsley, T., Weeks, L., Hempel, S., Akl, E. A., Chang, C., McGowan, J., Stewart, L., Hartling, L., Aldcroft, A., Wilson, M. G., Garritty, C., … Straus, S. E. (2018). PRISMA extension for scoping reviews (PRISMA-ScR): Checklist and explanation. *Annals of Internal Medicine*, *169*(7), 467–473. https://doi.org/10.7326/M18-0850
